# Comparing Claims Data to Stroke and Bleeding in the NCDR Left Atrial Appendage Occlusion Registry

**DOI:** 10.1016/j.jacadv.2025.102019

**Published:** 2025-07-24

**Authors:** Kamil F. Faridi, James V. Freeman, Yongfei Wang, Lucy Pereira, Sarah Zimmerman, Daniel J. Friedman, Richa Sharma, Angela Y. Higgins, Bobak J. Mortazavi, Joseph S. Ross, Harlan M. Krumholz, Robert W. Yeh, Jeptha P. Curtis

**Affiliations:** a Section of Cardiovascular Medicine, Department of Medicine, Yale School of Medicine, New Haven, Connecticut, USA; b Center for Outcomes Research and Evaluation, Yale New Haven Health, New Haven, Connecticut, USA; c Division of Cardiology, Duke University Medical Center and Duke Clinical Research Institute, Durham, North Carolina, USA; d Department of Neurology, Center for Brain and Mind Health, Yale University School of Medicine, New Haven, Connecticut, USA; e Division of Cardiology, Maine Health, Scarborough, Maine, USA; f Department of Computer Science and Engineering, Texas A&M University, College Station, Texas, USA; g Section of General Internal Medicine, Department of Medicine, Yale School of Medicine, New Haven, Connecticut, USA; h Richard A. and Susan F. Smith Center for Outcomes Research in Cardiology, Division of Cardiology, Beth Israel Deaconess Medical Center, Boston, Massachusetts, USA

**Keywords:** administrative claims, bleeding, left atrial appendage occlusion, outcomes, stroke

## Abstract

**Background:**

Claims data are increasingly used for postmarketing surveillance of therapies such as transcatheter left atrial appendage occlusion (LAAO), but their accuracy remains uncertain.

**Objectives:**

This study aimed to compare stroke and bleeding events in the National Cardiovascular Data Registry LAAO Registry with claims data.

**Methods:**

LAAO Registry data for patients aged ≥65 years were linked to 2016 to 2021 Medicare claims. Primary diagnosis International Classification of Diseases-Tenth Revision codes from inpatient hospitalizations were compared to adjudicated registry-reported stroke and major bleeding events after discharge, including estimation of sensitivity and positive predictive value of claims for identifying registry-reported events. Kappa statistics and incidence rates were also assessed.

**Results:**

Among 71,043 LAAO Registry patients, sensitivity and positive predictive value of claims were 60.8% and 50.5% for ischemic stroke (kappa 0.55), 42.7% and 50.5% for hemorrhagic stroke (kappa 0.46), 55.9% and 40.3% for gastrointestinal bleeding (GIB) (kappa 0.43), 62.2% and 38.0% for intracranial hemorrhage (kappa 0.47), and 20.4% and 10.0% for other major bleeding (kappa 0.12). Sensitivity and negative predictive values were >92% for all events. Two-year incidence rates were higher in claims vs registry data for ischemic stroke (2.5% vs 2.2%), GIB (6.8% vs 5.2%), intracranial hemorrhage (1.6% vs 1.1%), and other bleeding (3.1% vs 1.5%; *P* < 0.01 for all events), and lower for hemorrhagic stroke (0.4% vs 0.5%; *P* = 0.03).

**Conclusions:**

In the LAAO Registry, International Classification of Diseases-Tenth Revision codes have moderate agreement with stroke, GIB, and intracranial hemorrhage, and overestimate most event rates compared to adjudicated registry-reported events. Nonclaims-based methods are needed to ensure accurate assessment of clinical events in postmarketing surveillance.

Patients with atrial fibrillation are at increased risk of ischemic stroke, a leading cause of disability and death in the United States.^1^, ^2^, ^3^ Oral anticoagulation reduces the risk of ischemic stroke in patients with atrial fibrillation.^4^, ^5^, ^6^, ^7^, ^8^ However, up to 50% of these patients do not receive anticoagulation due to safety concerns such as high bleeding risk.^9^^,^^10^ In 2015, the Food and Drug Administration approved the Watchman device for transcatheter left atrial appendage occlusion (LAAO) for patients with atrial fibrillation and a clinical rationale for a nonpharmacologic alternative to warfarin.^11^ Following this approval, the Food and Drug Administration mandated its postmarketing surveillance, and Medicare required hospital participation in a dedicated national registry.^12^ The National Cardiovascular Data Registry (NCDR) LAAO Registry was designated for this requirement and therefore captures the vast majority of LAAO procedures in the United States, with follow-up data available out to 2 years after the procedure.^13^

To monitor the effectiveness and safety of new therapies such as the Watchman device, increased emphasis has been placed on using real-world data sources such as registry and billing claims for event detection.^14^^,^^15^ It is important that such data are accurate, easily obtainable, and low in cost. Using claims data can be less burdensome than site-based reporting requiring additional manual effort and could allow for longer follow-up. Medicare fee-for-service data may be particularly helpful, given that enrolled patients are typically followed through their lifespan.

Clinical events identified in claims data have frequently been used in observational studies, though their accuracy is often suboptimal. Older studies comparing outcomes in claims data with adjudicated outcomes have been limited to relatively small cohorts and International Classification of Diseases-9th Revision (ICD-9) billing codes, which are no longer used in the United States since adoption of International Classification of Diseases-10th Revision (ICD-10) codes in 2015.^16^, ^17^, ^18^, ^19^, ^20^, ^21^, ^22^, ^23^, ^24^, ^25^, ^26^, ^27^, ^28^, ^29^ ICD-10 includes more current medical terminology, different numbering, and more than 4 times the number of codes reported in ICD-9.^30^ However, many studies measuring performance of ICD-10 codes are from other countries and have uncertain generalizability to the United States.^28^^,^^29^^,^^31^, ^32^, ^33^, ^34^, ^35^ Studies of ICD-10 codes among U.S. patients have been limited by small samples, low numbers of participating centers, and/or lack of an alternative data source that can assess for false negatives, true negatives, overall event agreement, or incidence of events.^36^, ^37^, ^38^, ^39^, ^40^, ^41^

Given the limitations of existing evidence, large adjudicated national data sets such as the LAAO Registry linked to Medicare claims using ICD-10 codes may help better assess the accuracy of claims data for event ascertainment in current U.S. practice. The aim of this study was to compare acute stroke and bleeding events identified in administrative claims data to adjudicated registry-reported events in the LAAO Registry.

## Methods

### Data source

We used data from the NCDR LAAO Registry, including baseline demographic information, clinical characteristics, and clinical outcomes with event dates.^13^ All data are obtained from review of medical records and reported by participating sites on standardized online data collection forms at the time of initial hospitalization for LAAO and in follow-up at 45 days (±14 days), 6 months (−30 days to +60 days), 1 year (±60 days), and 2 years (±60 days) following discharge. Definitions for variables are provided by the NCDR as part of registry participation. LAAO Registry data are audited annually at 48 randomly selected sites nationwide. The most recent LAAO Registry audit demonstrated an agreement rate of 94% when comparing registry-reported data with source document review, with 94% of sites scoring above 92% agreement. The NCDR LAAO Registry data have been linked with Centers for Medicare & Medicaid Services (CMS) claims data using direct and indirect patient identifiers. Linked Medicare data contain administrative claims data from all inpatient hospitalizations for Medicare fee-for-service beneficiaries. Mortality data are obtained from the CMS Master Beneficiary Summary file. This study was approved by the Yale University School of Medicine Institutional Review Board without requiring informed consent because all data were deidentified.

### Study population

We included all patients in the NCDR LAAO Registry ≥65 years (the age of eligibility for Medicare) who were discharged alive from the index LAAO hospitalization between January 1, 2016, and December 31, 2021, with data linked to Medicare fee-for-service claims. Patients who were not discharged alive from the index hospitalization and those who could not be linked to Medicare fee-for-service claims were excluded (Supplemental Figure 1). All adults enrolled in Medicare Advantage or other commercial insurance would not have Medicare fee-for-service claims data available and were thus excluded. All patients were required to have postdischarge follow-up data available, and thus all had at least a first follow-up visit in the 45-day time window. Patients were followed from the day after index LAAO discharge until whichever date came first among the following: the most recent site-reported registry data collection form date (up to 2 years after index LAAO), death, or the end of the study period (December 31, 2021). Since the LAAO Registry cannot report any events past the date of the last follow-up visit, events in claims data were not evaluated past that date for any given patient. For example, if a patient only had follow-up through the 1-year visit, no events were evaluated past the date reported for that visit in the registry. Since this study only assessed events occurring after discharge, all events occurring during the index LAAO hospitalization were also excluded from analysis.

### Data definitions and clinical outcomes

The primary outcomes for this study were acute stroke and major bleeding events occurring after LAAO hospitalization discharge. Adjudicated stroke and major bleeding events were used for analyses based on previously developed automated algorithms applied to LAAO Registry data, in conjunction with manual adjudication for select events meeting specific criteria (additional details provided in Supplemental Methods).^42^ These algorithms have previously demonstrated >90% agreement with clinical events committee adjudication of medical records.^42^ Ischemic stroke in the LAAO Registry is defined as a focal neurologic deficit of sudden onset as diagnosed by a neurologist, lasting >24 hours and caused by ischemia. Hemorrhagic stroke in the LAAO Registry is defined as an acute episode of focal or global cerebral or spinal dysfunction caused by intraparenchymal, intraventricular, or subarachnoid hemorrhage; subdural hematomas are excluded from this outcome and counted separately as intracranial hemorrhage. Undetermined cause of stroke can also be reported on the LAAO Registry data collection form and was included in analyses assessing total stroke.

Major bleeding events were evaluated based on adjudicated registry data and included intracranial hemorrhage (defined in the LAAO Registry as either a hemorrhagic stroke or intracranial hemorrhage other than hemorrhagic stroke), major gastrointestinal bleeding (GIB), and all other major bleeding events defined in the LAAO Registry (vascular complications and access site bleeding, hematoma, retroperitoneal bleeding, other nonintracranial hemorrhage, hemothorax, and pericardial effusion requiring intervention). All-cause mortality was also evaluated.

Stroke and bleeding events after hospital discharge were identified in claims data using ICD-10 diagnosis codes diagnosis codes based on use in prior studies and clinical plausibility (Supplemental Tables 1 and 2).^30^^,^^33^^,^^36^, ^37^, ^38^, ^39^, ^40^, ^41^^,^^43^, ^44^, ^45^, ^46^, ^47^ All patients in this study were ≥65 years of age, and therefore pregnancy-associated bleeding codes were not included. Events in claims were defined using ICD-10 codes reported as primary (one per hospitalization) or secondary (up to 24 per hospitalization) discharge diagnosis codes from inpatient hospitalizations. All-cause mortality is reported in the LAAO Registry as well as in Medicare data.

### Statistical analysis

Similar to prior studies comparing claims data with adjudicated events,^16^, ^17^, ^18^, ^19^^,^^22^, ^23^, ^24^^,^^27^, ^28^, ^29^^,^^33^^,^^39^^,^^41^^,^^43^^,^^47^ we assessed concordance of clinical events in the LAAO Registry with events in Medicare claims data. Since the LAAO Registry includes site-reported data based on prespecified event definitions and direct review of medical records, random auditing to assess data accuracy, a validated automated adjudication algorithm,^42^ and additional independent manual adjudication of events meeting certain criteria (Supplemental Methods), stroke and bleeding events in the registry were treated as the reference standard for clinical events. In primary analyses, events identified in claims data were considered a match with a registry event (a “true positive”) if the associated inpatient hospitalization with a designated primary diagnosis code was within 14 days of the registry event date. If multiple registry events occurred during the dates of a hospitalization with a relevant primary diagnosis code, this was counted as only one true positive. If a hospitalization meeting criteria for an event was identified in claims outside the 14-day window, it was considered a “false positive.” If multiple hospitalization events occurred within the 14-day window of a registry event, only one true positive was counted and all other events in claims were counted as false positives. True positives, true negatives, false positives, and false negatives were used to calculate sensitivity, specificity, positive predictive value (PPV), and negative predictive value (NPV) for each clinical outcome. To assess event agreement without using a reference standard, we also determined kappa statistics for each event type. Initial and recurrent events were included in all calculations, and performance of specific ICD-10 codes was reported based on frequency. Similar analyses were performed for mortality using a 14-day matching window. Since mortality in Medicare data has been shown to be equivalent to death adjudicated by Clinical Event Committees from multiple large clinical trials,^27^^,^^43^ death in Medicare data was considered the reference standard.

In secondary analyses, comparability of claims data and registry data was assessed by adjusting event definitions. In order to evaluate how performance may depend on reported event dates, the window considered as a match between a registry and claims event was narrowed to 7 days and extended to 30 days. Events in claims data were also adjusted to include secondary diagnosis codes from hospitalizations in addition to primary diagnosis codes. For GIB and other major bleeding events, we also included an ICD-10-Procedure Coding System code for red blood cell transfusion. Comparisons were also stratified by sex. We further evaluated specificity and PPV of claims data for identifying registry events occurring at the hospital where the LAAO procedure was performed. Similar analyses were performed for events at university hospitals (98 sites) and private/community hospitals (561 sites). Sensitivity and NPV could not be determined in these hospital-based supplemental analyses because the site of clinical events was available in claims data but not registry data.

In additional analyses, we used time-to-event analyses with Kaplan-Meier methods to estimate cumulative incidence of postdischarge stroke and major bleeding in LAAO Registry and claims data with up to 2 years of follow-up, accounting for competing risk of death using the Fine-Gray method.^48^ Incidence rates in registry and claims data for each outcome were compared by using an indicator for data source (registry or claims) in the model. HRs using the data source indicator were also determined using unadjusted competing risk models with Cox regression. We additionally tested proportional hazard assumptions for individual events in registry vs claims data. Two-sided *P* values < 0.05 were considered statistically significant. All analyses were completed using SAS version 9.4 (SAS Institute) at the Yale/Yale New Haven Health Center for Outcomes Research and Evaluation (CORE) Data Analytic Center.

## Results

### Patient characteristics

The study included postdischarge follow-up data from 71,043 patients at 671 sites in the LAAO Registry with data linked to Medicare (Supplemental Figure 1). The median follow-up was 1.0 (IQR: 0.5-2.0) years. Similar to prior investigations from the LAAO Registry,^13^^,^^42^ the cohort consisted of older adults with a mean age of 77.5 (SD: 6.5) years, a mean CHA_2_DS_2_-VASc score of 3.0 (SD: 1.3), and a mean HAS-BLED score of 3.0 (SD: 1.1). Overall, 41.8% were women, 22.1% had a history of prior stroke (5.2% with ischemic stroke and 14.2% with hemorrhagic stroke), and 63.5% had a prior history of major bleeding (59.4% with a prior GIB). Clinical characteristics of LAAO Registry participants included in the study cohort were similar to participants ≥65 years of age who otherwise met the inclusion criteria but were excluded due to inability to link with Medicare fee-for-service claims data (Table 1), suggesting broad generalizability of the study cohort.

**Table 1:** LAAO Registry Patient Clinical Characteristics

	Study Cohort With Linked Medicare Data (n = 71,043)	Excluded Cohort ≥65 Years Without Linked Medicare Data (n = 49,400)	Standardized Difference
Age, y	77.5 (6.5)	77.4 (6.6)	2.5
Female	29,673 (41.8)	20,809 (42.1)	0.7
Race			
White	67,469 (95.0)	45,541 (92.2)	11.6
Black	2,075 (2.9)	2,514 (5.1)	11.3
Asian	767 (1.1)	711 (1.4)	3.3
Other	532 (0.8)	409 (0.8)	0.9
Hispanic ethnicity	2,157 (4.4)	1,568 (2.2)	2.9
Body mass index, kg^2^/m^2^	29.7 (9.6)	29.8 (10.3)	1.5
CHA_2_DS_2_-VASc score	3.0 (1.3)	3.0 (1.3)	4.9
Congestive heart failure	26,190 (36.9)	19,308 (39.1)	4.6
Cardiomyopathy	13,722 (19.4)	9,946 (20.2)	2.1
Hypertension	65,218 (91.8)	45,514 (92.2)	1.2
Diabetes mellitus	24,808 (34.9)	18,298 (37.1)	4.4
Prior stroke	15,696 (22.1)	11,618 (23.5)	3.4
Prior transient ischemic attack	9,145 (12.9)	6,383 (12.9)	0.1
Prior thromboembolic event	11,300 (15.9)	7,988 (16.2)	0.7
Coronary artery disease	34,315 (48.3)	23,919 (48.4)	0.2
Peripheral artery disease	8,585 (12.1)	5,769 (11.7)	1.3
Chronic lung disease	14,586 (20.6)	10,654 (21.6)	2.5
Obstructive sleep apnea	20,168 (28.4)	12,976 (26.3)	4.8
Glomerular filtration rate, mL/min/1.73 m^2^	66 (23)	66 (23)	0.7
HAS-BLED score	3.0 (1.1)	3.0 (1.1)	3.1
Clinically relevant prior bleeding	45,038 (63.5)	32,274 (65.4)	4.0
Intracranial	7,099 (15.8)	5,148 (16.0)	1.4
Epistaxis	4,388 (9.8)	3,016 (9.4)	0.3
Gastrointestinal	26,607 (59.4)	19,622 (61.1)	4.7
Other	10,635 (23.7)	7,067 (22.0)	1.9
Increased fall risk	29,430 (41.5)	20,626 (41.9)	0.7
Arrhythmia history			
Atrial fibrillation type			
Paroxysmal	40,947 (57.6)	28,086 (56.9)	1.6
Persistent (>7 days)	14,658 (20.6)	10,245 (20.7)	0.3
Long-standing persistent (>1 year)	5,047 (7.1)	3,531 (7.1)	0.2
Permanent	9,966 (14.0)	7,223 (14.6)	1.7
Atrial flutter			
Typical	7,025 (9.9)	4,615 (9.3)	1.9
Atypical	3,035 (4.3)	1,909 (3.9)	2.1
Prior Afib termination attempt	30,307 (42.7)	20,263 (41.1)	3.3

Data are shown for LAAO Registry patients aged ≥65 years. Standardized differences between the study cohort and excluded cohort are shown (values >10 are considered significant).

LAAO = left atrial appendage occlusion.

### Comparisons of stroke events

Among 1,308 total postdischarge stroke events reported in the LAAO Registry for Medicare beneficiaries with linked data, 998 (76.3%) were ischemic strokes, 241 (18.4%) hemorrhagic strokes, and 69 (5.3%) undetermined strokes. In primary analyses for claims data, there were 1,201 ischemic strokes and 204 hemorrhagic strokes. The sensitivity and PPV of claims data for identifying stroke events in the LAAO Registry was 58.1% (95% CI: 55.4% to 60.8%) and 54.1% (95% CI: 51.9% to 56.3%) for all stroke events, 60.8% (95% CI: 57.8% to 63.9%) and 50.5% (95% CI: 48.2% to 52.9%) for ischemic stroke events, and 42.7% (95% CI: 36.5% to 49.0%) and 50.5% (95% CI: 44.4% to 56.6%) for hemorrhagic stroke events, respectively. The specificity and NPV for all types of stroke events were >99%, reflecting the relatively low incidence of these events following hospital discharge after transcatheter LAAO. There was moderate agreement between claims data and the LAAO Registry for all stroke events (kappa statistic 0.55; 95% CI: 0.53-0.57), with greater agreement for ischemic stroke events compared to hemorrhagic stroke events (Table 2). PPV of specific ICD-10 codes used for stroke diagnosis is shown in Supplemental Table 3. Frequency of nonstroke ICD-10 codes used as the primary diagnosis for hospitalizations that occurred at the time of a registry-reported stroke (“false negatives”) is shown in Supplemental Table 4.

**Table 2:** Stroke Events in Claims Data Compared to LAAO Registry Data

Present in Registry (n)	Present in Claims Data (n)			Sensitivity, % (95% CI)	Specificity, % (95% CI)	PPV, % (95% CI)	NPV, % (95% CI)	Kappa Coefficient (95% CI)
	Yes	No	Total					
All stroke events								
Yes	760	548	1,308	58.1 (55.4-60.8)	99.1 (99.0-99.2)	54.1 (51.9-56.3)	99.2 (99.2-99.3)	0.55 (0.53-0.57)
No	645	69,337	69,982					
Total	1,405	69,885	71,290					
Ischemic stroke events								
Yes	607	391	998	60.8 (57.8-63.9)	99.2 (99.1-99.2)	50.5 (48.2-52.9)	99.4 (99.4-99.5)	0.55 (0.52-0.57)
No	594	69,659	70,253					
Total	1,201	70,050	71,251					
Hemorrhagic stroke events								
Yes	103	138	241	42.7 (36.5-49.0)	99.9 (99.9-99.9)	50.5 (44.4-56.6)	99.8 (99.8-99.8)	0.46 (0.40-0.52)
No	101	70,778	70,879					
Total	204	70,916	71,120					

Events in Medicare claims data were determined using ICD-10 codes reported as the primary diagnosis for inpatient hospitalizations. Registry events were reported by sites during follow-up in the LAAO Registry after index LAAO hospitalization discharge and included previously validated adjudication algorithms. Registry events were used as the reference standard for sensitivity, specificity, PPV, and NPV. Analyses were performed at the level of events, with inclusion of all initial and recurrent events.

NPV = negative predictive value; PPV = positive predictive value; other abbreviation as in Table 1.

In secondary analyses, inclusion of secondary diagnosis codes improved sensitivity of claims (67.0% [95% CI: 64.4% to 69.5%] for all stroke, 70.3% [95% CI: 67.5% to 73.2%] for ischemic stroke, and 53.9% [95% CI: 47.7% to 60.2%] for hemorrhagic stroke), though PPV decreased (47.8% [95% CI: 46.0% to 49.6%] for all stroke, 44.8% [95% CI: 42.9% to 46.7%] for ischemic stroke, and 34.8% [95% CI: 30.9% to 38.6%] for hemorrhagic stroke). Using an alternative match window of 7 or 30 days, excluding patients with a prior history of stroke, and stratification by sex did not appreciably impact performance of claims data in identifying stroke events (Supplemental Tables 5 and 6).

### Comparisons of major bleeding events

Among all major postdischarge major bleeding events reported in the LAAO Registry, 3739 (71.1%) were GIB, 555 (10.5%) were intracranial hemorrhage, and 967 (18.4%) were other bleeding events. In primary analyses for claims data, there were 5,188 GIB events, 907 intracranial hemorrhage events, and 1,974 other bleeding events. The sensitivity, specificity, PPV, and NPV of claims data for identifying major bleeding in the LAAO Registry are shown in Table 3. Claims data had modest sensitivity and PPV for GIB (55.9% [95% CI: 54.3% to 57.5%] and 40.3% [95% CI: 39.2% to 41.3%]) and intracranial hemorrhage (62.2% [95% CI: 58.1% to 66.2%] and 38.0% [95% CI: 35.6% to 40.5%]), but very low sensitivity and PPV for all other bleeding events (20.4% [95% CI: 17.8% to 22.9%] and 10.0% [95% CI: 8.8% to 11.2%], respectively). Overall agreement between claims and LAAO Registry data was moderate for GIB (kappa statistic 0.43; 95% CI: 0.42-0.45) and intracranial hemorrhage (kappa 0.47; 95% CI: 0.44-0.50), and poor for all other bleeding events (kappa 0.12; 95% CI: 0.10-0.13). PPV of specific ICD-10 codes used for bleeding events is shown in Supplemental Table 3. The most common nonbleeding ICD-10 codes used for primary diagnoses of hospitalizations occurring at the time of a registry-reported bleeding event are shown in Supplemental Table 4.

**Table 3:** Bleeding Events in Claims Data Compared to LAAO Registry Data

Present in Registry (n)	Present in Claims Data (n)			Sensitivity, % (95% CI)	Specificity, % (95% CI)	PPV, % (95% CI)	NPV, % (95% CI)	Kappa Coefficient (95% CI)
	Yes	No	Total					
All Major Bleeding Events								
Yes	3,010	2,243	5,253	57.3 (56.0-58.6)	92.6 (92.4-92.8)	37.4 (36.5-38.2)	96.6 (96.5-96.7)	0.40 (0.39-0.41)
No	5,046	63,410	68,546					
Total	8,056	65,653	73,709					
Intracranial hemorrhage events								
Yes	345	210	555	62.2 (58.1-66.2)	99.2 (99.1-99.3)	38.0 (35.6-40.5)	99.7 (99.7-99.7)	0.47 (0.44-0.50)
No	562	70,120	70,682					
Total	907	70,330	71,237					
Gastrointestinal bleeding events								
Yes	2,089	1,650	3739	55.9 (54.3-57.5)	95.5 (95.4-95.7)	40.3 (39.2-41.3)	97.6 (97.5-97.7)	0.43 (0.42-0.45)
No	3,099	65,967	69,066					
Total	5,188	67,617	72,805					
Other major bleeding events								
Yes	197	770	967	20.4 (17.8-22.9)	97.5 (97.4-97.6)	10.0 (8.8-11.2)	98.9 (98.9-98.9)	0.12 (0.10-0.13)
No	1,777	68,668	70,445					
Total	1,974	69,438	71,412					

Events in Medicare claims data were determined using ICD-10 codes reported as the primary diagnosis for inpatient hospitalizations. Registry events were reported by sites during follow-up in the LAAO Registry after index LAAO hospitalization discharge and included previously validated adjudication algorithms. Registry events were used as the reference standard for sensitivity, specificity, PPV, and NPV. Analyses were performed at the level of events, with inclusion of all initial and recurrent events.

Abbreviations as in Tables 1 and 2.

Results of secondary analyses for bleeding are shown in Supplemental Tables 6 and 7. Similar to stroke events, performance of claims data was similar regardless of whether the match window varied between 7 and 30 days, while inclusion of secondary diagnosis codes increased sensitivity (72.7% [95% CI: 71.2% to 74.1%] for GIB, 72.6% [95% CI: 68.9% to 76.3%] for intracranial hemorrhage, and 50.2% [95% CI: 47.1% to 53.4%] for other bleeding events) but decreased PPV (31.8% [95% CI: 31.1% to 32.5%] for GIB, 31.7% [95% CI: 29.9% to 33.5%] for intracranial hemorrhage, and 3.5% [95% CI: 3.1% to 3.7%] for other bleeding). Inclusion of Procedure Coding System codes for blood transfusion increased sensitivity and PPV of bleeding events when only primary diagnosis codes were used, but did not substantially impact these measures when secondary diagnosis codes were also included (Supplemental Table 7). Performance was generally similar after excluding patients with prior bleeding (Supplemental Table 7). Performance of claims data for bleeding was also similar between men and women (Supplemental Table 6).

### Comparisons of events based on hospital site

Based on the number of events matched in both registry and claims data, the vast majority of postdischarge stroke admissions occurred at the hospital where the LAAO was performed. For example, among 686 matched ischemic stroke events using primary and secondary diagnosis codes, 535 (80.0%) occurred at the LAAO hospital. The PPV of primary diagnosis codes for ischemic and hemorrhagic stroke was higher at LAAO hospitals (55.8% [95% CI: 52.7% to 58.8%] and 52.9% [95% CI: 45.7% to 60.2%]) compared to non-LAAO hospitals (39.7% [95% CI: 35.1% to 44.3%] and 46.2% [95% CI: 33.0% to 59.3%], respectively). The trend was similar when secondary diagnosis codes were included for stroke (Supplemental Table 8). Higher PPV for ischemic stroke events was observed at private/community hospitals (51.7%, 95% CI: 49.1% to 54.3%) compared to university hospitals (45.0%, 95% CI: 39.5% to 50.6%), however the proportion of ischemic strokes at University hospitals was relatively small (12.7% of all ischemic strokes in the LAAO Registry); PPV for hemorrhagic stroke at university and private/community hospitals was similar (Supplemental Table 9).

Similar to stroke, more matched bleeding events occurred at LAAO hospitals (71.7% of all matched major bleeding events using primary and secondary codes). Also similar to stroke events, PPV for bleeding in claims data was higher at LAAO vs non-LAAO hospitals using either primary codes (41.6% [95% CI: 40.5% to 42.7%] vs 31.2% [95% CI: 29.6% to 32.8%]) or primary and secondary codes (24.8% [95% CI: 24.2% to 25.4%] vs 17.5% [95% CI: 16.7% to 18.3%]), whereas differences between university and private/community hospitals were smaller (Supplemental Tables 10 and 11).

### Comparisons of cumulative incidence of stroke and major bleeding

**Figure 1:**
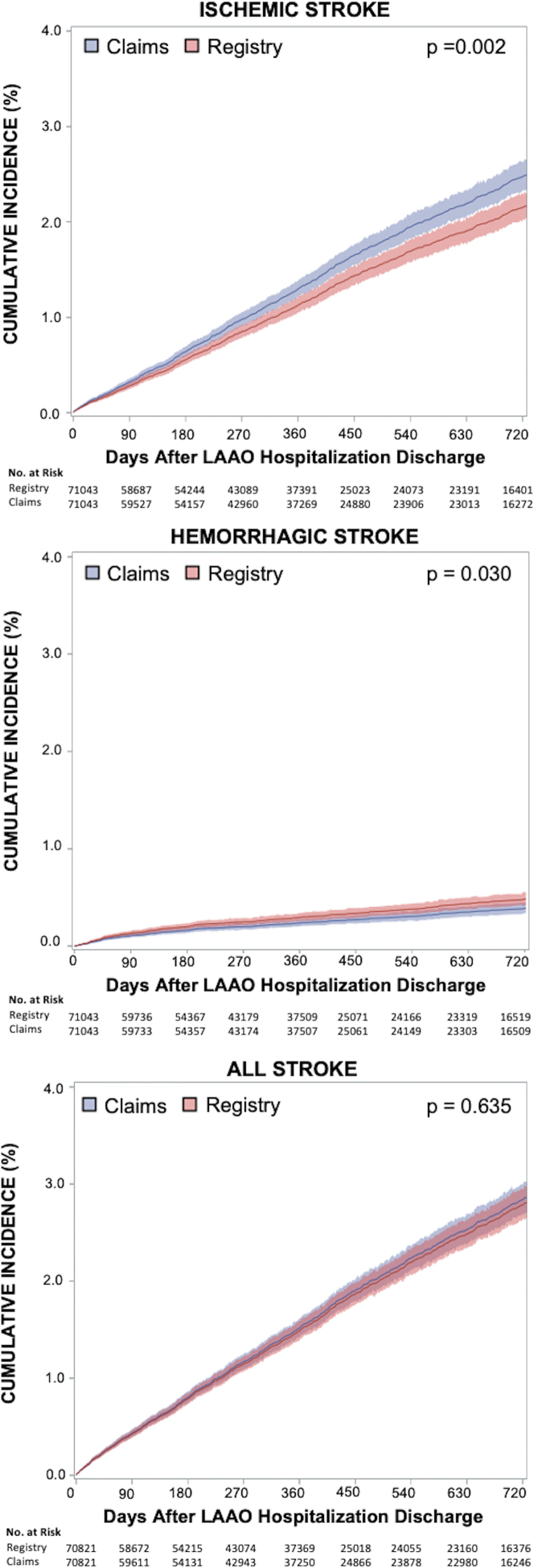
**Incidence of Stroke Events in LAAO Registry vs Medicare Claims** Cumulative incidence of ischemic stroke, hemorrhagic stroke, and all stroke from time of discharge up to 2 years after the index LAAO hospitalization are shown. The curves for Claims represent hospitalizations with primary diagnosis codes for each event in Medicare claims data. The Registry curves represent events identified in the NCDR LAAO Registry. Boundaries for 95% CIs are shown. LAAO = left atrial appendage occlusion; NCDR = National Cardiovascular Data Registry.

Cumulative incidence of ischemic stroke at 2 years following LAAO hospitalization discharge was higher in claims data than in registry data (2.47% vs 2.15%; HR: 1.15; 95% CI: 1.05-1.26; *P* = 0.002), whereas incidence of hemorrhagic stroke was slightly lower in claims data (0.39% vs 0.48%; HR: 0.81; 95% CI: 0.66-0.98; *P* = 0.030). When ischemic and hemorrhagic stroke events were combined to assess incidence of all stroke, there was no significant difference between registry and claims data (Figure 1, Supplemental Table 12).

**Figure 2:**
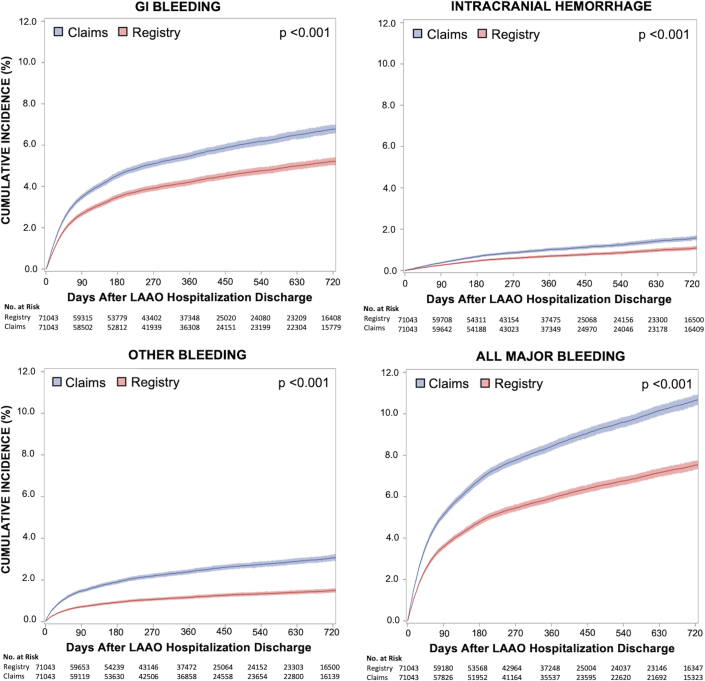
**Incidence of Bleedings Events in LAAO Registry vs Medicare Claims** Cumulative incidence of major GI bleeding, intracranial hemorrhage, other major bleeding and all major bleeding from time of discharge up to 2 years after the index LAAO hospitalization are shown. The curves for Claims represent hospitalizations with primary diagnosis codes for each event in Medicare claims data. The Registry curves represent events identified in the NCDR LAAO Registry. Boundaries for 95% CIs are shown. GI = gastrointestinal; other abbreviations as in Figure 1.

For major bleeding events, 2-year cumulative incidence of events in claims data was higher for GIB (6.77% vs 5.21%; HR: 1.31; 95% CI: 1.25-1.37; *P* < 0.001), intracranial hemorrhage (1.56% vs 1.08%; HR: 1.46; 95% CI: 1.30-1.63; *P* < 0.001), and other major bleeding (3.06% vs 1.49%; HR: 2.07; 95% CI: 1.90-2.25; *P* < 0.001). Incidence of combined major bleeding events was consequently also higher in claims data (Figure 2, Supplemental Table 12). All individual events demonstrated proportional hazards except for other major bleeding, though overall agreement between registry and claims for other bleeding was similar in early vs late follow-up (Supplemental Table 13).

### Comparisons of all-cause mortality

Using CMS data as the reference standard for all-cause mortality,^27^^,^^43^ the LAAO Registry was extremely accurate in identifying death with a sensitivity of 93.0% (95% CI: 91.4% to 94.6%) and a specificity of >99.9%, with overall excellent agreement between the 2 data sources (kappa statistic 0.96; 95% CI: 0.95-0.97) (Supplemental Table 14).

## Discussion

In this study of over 70,000 Medicare patients in the NCDR LAAO Registry, we found that contemporary claims data with ICD-10 codes have moderate agreement with adjudicated registry-reported stroke, GIB and intracranial hemorrhage, and poor agreement with other major bleeding events. Claims data significantly overestimates the cumulative incidence of ischemic stroke and major bleeding events and underestimates hemorrhagic stroke (Central Illustration). These findings highlight the limitations of using claims data for postmarketing surveillance of stroke and bleeding outcomes in the United States.

**Central Illustration:**
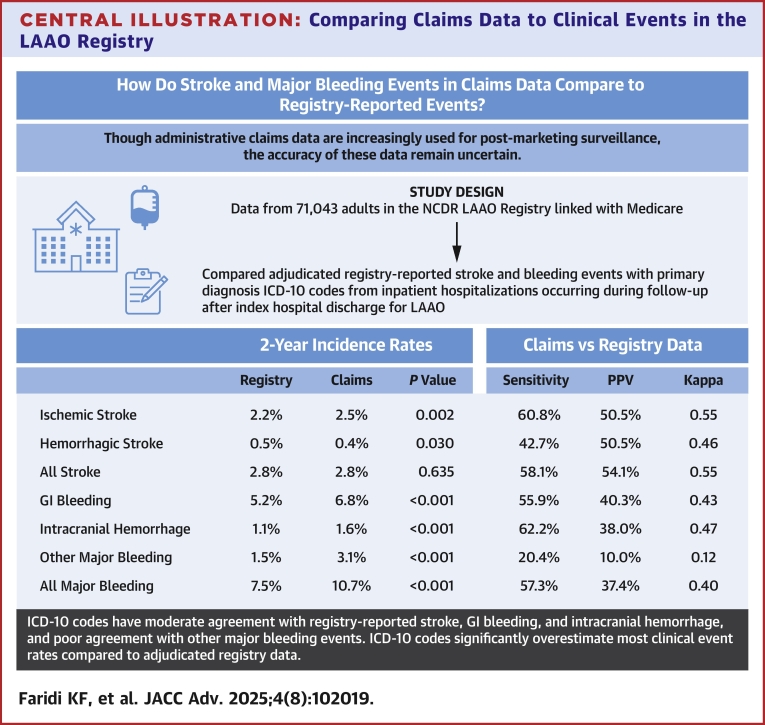
**Comparing Claims Data to Clinical Events in the LAAO Registry** All results are from NCDR LAAO Registry data for patients aged ≥65 years linked to 2016 to 2021 Medicare claims. All events were evaluated after discharge from the index LAAO hospitalization with up to 2 years of follow-up. Incidence rates were determined using Kaplan-Meier methods, and *P* values represent differences between registry and claims using an indicator for data source in the models. Sensitivity and PPV were determined using registry-reported events as the reference standard, and agreement was assessed with kappa statistics. ICD-10 = International Classification of Diseases-Tenth Revision; PPV = positive predictive value; other abbreviations as in Figures 1 and 2.

Our study represents one of the largest and most comprehensive studies performed for patients with data available in claims and a separate data source with adjudicated events. Earlier U.S. studies have primarily evaluated ICD-9 codes which are no longer in use and differ from current ICD-10 definitions^16^, ^17^, ^18^, ^19^, ^20^, ^21^, ^22^, ^23^, ^24^, ^25^, ^26^, ^27^, ^28^, ^29^ and are therefore not relevant to contemporary practice. Furthermore, the LAAO Registry was uniquely developed for monitoring clinically important events after widespread adoption of LAAO in the United States. Our study can therefore uniquely inform postmarketing surveillance using real-world data sources.

We showed that claims data have moderate agreement with registry-reported data for stroke. Most registry-reported strokes were identified with primary diagnoses in claims data (sensitivity 58%), though the PPV for a stroke event in claims was only 54%. Claims data had greater sensitivity and overall agreement with registry-reported ischemic strokes compared to hemorrhagic strokes. Two studies based on chart review of stroke events in U.S. claims data reported PPV ranging from 72% to 100% for similar ICD-10 codes, though these studies were much smaller and did not have a separate nonclaims data source, and therefore could not evaluate sensitivity, overall event agreement, or incidence.^36^^,^^39^ In more comprehensive analyses using data from the ARIC (Atherosclerosis Risk in Communities) and REGARDS (Reasons for Geographic and Racial Differences in Stroke) prospective studies, Medicare claims had a sensitivity of 70.7% to 81.8% and a PPV of 64.7% to 65.2% for identifying adjudicated events.^40^

Notably, all these studies demonstrated higher performance for claims data than what we observed. The prior prior investigations of ARIC and REGARDS also found no statistically significant differences in adjudicated vs claims-based ischemic stroke incidence rates, though strokes were numerically higher in claims. In contrast, we found that incidence of ischemic stroke was significantly higher in claims data whereas hemorrhagic stroke incidence was lower, and combining stroke types resulted in no significant difference in total stroke incidence. Importantly, our study included more than 1,300 total stroke events, whereas the prior U.S. studies evaluating similar ICD-10 codes included 150 or fewer events from each data source.

Our investigation also showed that ICD-10 codes have moderate agreement with registry-reported GIB and intracranial hemorrhage. Primary diagnosis ICD-10 codes identified most registry GIB events (sensitivity 56%) and intracranial hemorrhages (sensitivity 62%), though less than half of claims-based events were reported in the registry. Compared to registry data, the 2-year incidence of GIB was consequently 33% higher and the incidence of intracranial hemorrhage was 46% higher in claims data. Sensitivity and PPV of primary diagnosis ICD-10 claims was poor, and 2-year incidence of other bleeding was more than twice that of the registry.

In a smaller study of Medicare patients based on chart reviews of 397 bleeding events using primary diagnosis ICD-10 codes, PPV for all types of bleeding was reported to be over 80%, which was much higher than in our study.^38^ Unlike our study which had an alternative nonclaims data source, sensitivity could not be directly calculated in this prior study but was estimated with a sampling methodology to be 53% for GIB, 71% for intracranial hemorrhage, and 24% for other bleeding, similar to what we observed. Another single-center U.S. study reported a PPV of 63% specifically for intracranial hemorrhage, which was similar to what we observed, though the sensitivity was higher at 89%.^37^ Cumulative incidence in these 2 studies could not be determined. Our study also included more than 5,200 registry-reported major bleeding events at nearly 700 hospitals, representing more than a 10-fold greater number of events and more than a 100-fold greater number of hospitals compared to these prior investigations.

In supplemental analyses, we also demonstrated that inclusion of secondary diagnosis codes increased the sensitivity for identifying registry-based events but decreased PPV across all outcomes. Inclusion of red blood cell transfusions and varying the event window between 7 and 30 days did not have a significant impact compared to diagnosis codes. Performance of claims was also similar for men and women. Though diagnosis codes in claims may reflect prior rather than acute events, we found that performance was comparable even after excluding patients with prior stroke or bleeding. The PPV of claims data was also higher for events occurring at hospitals where the LAAO was performed. Lastly, we demonstrated that death reported in the LAAO Registry is highly accurate compared to death recorded in CMS data.

Our findings have several implications. Given that accuracy of event ascertainment from claims data are limited compared to events identified with adjudication methods in the LAAO Registry, our data suggest that claims should not be completely relied upon as the sole data source for stroke or major bleeding in postmarketing surveillance studies. Given their relatively modest PPV (<60% for all outcomes and all definitions we analyzed), they will likely overestimate the total number of events and cumulative incidence of outcomes, particularly for ischemic stroke and bleeding. Since performance of claims for major bleeding other than GIB and intracranial hemorrhage was particularly poor, our findings suggest they should not be included in studies without additional rigorous ascertainment of such bleeding events. Even so, claims data may have significant utility for screening potential events since they captured ≥70% of most outcomes when primary and secondary diagnosis codes were used. A possible event in claims data could prompt further assessment of medical records, for example.

### Study Limitations

Importantly, though we used the LAAO Registry as the reference standard for analytic purposes in this study, it does not necessarily represent the optimal strategy for postmarketing surveillance. Strengths of the registry include regularly scheduled follow-up visits, standardized data collection forms and event definitions across sites, site reporting using review of medical records, and annual data auditing routinely demonstrating >92% agreement with manual source document review. However, it is still possible that events meeting registry definitions and identified in claims data may have occurred but were not reported at follow-up visits. This could be particularly true for events at hospitals where the LAAO was not performed, given the lower PPV for claims we observed at these sites. Furthermore, a clinical events committee did not review all site-reported events. Though review of medical records for all events was not possible, the LAAO Registry uses an adjudication process that includes an automated algorithm and manual adjudication of events meeting prespecified criteria. This has been validated against manual clinical events committee adjudication with >90% agreement for major adverse events based on sampled data.^42^ In addition, since claims data did not capture a substantial portion of events reported in the LAAO Registry, observed sensitivities would likely be similar even if events were missed by the registry. Though beyond the scope of our available data, future efforts should focus on combining multiple data sources including registry data, claims data, and electronic health records which may allow for more comprehensive and/or accurate event identification.

This study has several additional limitations. Our analysis only included patients with atrial fibrillation who underwent LAAO, reflecting an older population with more comorbidities and higher rates of events that may not be generalizable to the general population. We also only had data available for patients enrolled in Medicare fee-for-service and could not account for patients switching to Medicare Advantage or other insurance. However, this was likely infrequent in our cohort since most of our event data was within 1 year of being enrolled in Medicare fee-for-service and a prior study demonstrated that <10% of such patients switch to Medicare Advantage per year.^49^ Registry data were based on specific reporting protocols and definitions for the LAAO Registry, though definitions are similar to those used in other registries and clinical trials. Our analysis was also limited to ICD-10 codes from inpatient hospitalizations, which may not capture less severe events from emergency department visits or observation stays.

## Conclusions

In this nationwide study of Medicare patients in the NCDR LAAO Registry, we found that ICD-10 codes from inpatient hospitalizations have moderate agreement with stroke, GIB, and intracranial hemorrhage compared to registry-reported data utilizing adjudication methods. Claims data have poor agreement for other major bleeding events and overestimate event rates for most stroke and bleeding outcomes. These findings indicate additional event ascertainment methods are likely needed to ensure accurate assessment of clinical events in postmarketing surveillance efforts.

Perspectives **COMPETENCY IN MEDICAL KNOWLEDGE:** Administrate claims data with ICD-10 codes are commonly used for postmarketing surveillance of cardiovascular therapies such as transcatheter LAAO, though accuracy of these data remains uncertain. This study linked the NCDR LAAO Registry with Medicare claims data and showed that ICD-10 codes from inpatient hospitalizations have moderate agreement with registry-reported stroke, GIB, and intracranial hemorrhage, and poor agreement with other major bleeding events. Claims data significantly overestimated the cumulative incidence of ischemic stroke and most major bleeding events.

**TRANSLATIONAL OUTLOOK:** This study demonstrates that claims data using contemporary ICD-10 codes have modest performance and overestimate most events compared to adjudicated follow-up data reported in the LAAO Registry. These findings indicate additional event ascertainment methods are likely needed to ensure accurate assessment of clinical events in postmarketing surveillance efforts.

## Funding support and author disclosures

Dr Faridi has received research funding from the 10.13039/100000050NIH/NHLBI (K23HL161424). Dr Freeman has received research grants from the 10.13039/100000050National Heart, Lung, and Blood Institute and the 10.13039/100005485American College of Cardiology National Cardiovascular Data Registry and consulting/advisory board fees from Boston Scientific, Biosense Webster, Medtronic, and PaceMate and equity in PaceMate. Dr Wang has received compensation for performing data analytic services for the American College of Cardiology. Dr Pereira has received compensation for performing data analytic services for the American College of Cardiology. Dr Zimmerman has received compensation for performing data analytic services for the American College of Cardiology. Dr Friedman has received research grants from Abbott, American Heart Association, Biosense Webster, Boston Scientific, Medtronic, Merit Medical, National Cardiovascular Data Registry, Philips, and National Institutes of Health, and consulting fees from Abbott, NI Medical, Microport, and Sanofi. Dr Sharma has received grants from the 10.13039/100000065National Institutes of Health/National Institute of Neurological Disorders and Stroke and having a pending patent for Methods of Training an Algorithm to Predict Ischemic Stroke Etiology. Dr Mortazavi has received research funding from the 10.13039/100000001NSF (IIS#2014475) and the 10.13039/100000002NIH (R21EB028486, R01HL151240, R01EB028106, and R01HL142765). Dr Ross currently has received research support through Yale University from Johnson and Johnson to develop methods of clinical trial data sharing, from the Food and Drug Administration for the Yale-Mayo Clinic Center for Excellence in Regulatory Science and Innovation (10.13039/100008935CERSI) program (U01FD005938), from the 10.13039/100000133Agency for Healthcare Research and Quality (R01HS022882), and from Arnold Ventures; formerly received research support from the Medical Device Innovation Consortium as part of the National Evaluation System for Health Technology (NEST) and from the 10.13039/100000050National Heart, Lung, and Blood Institute of the National Institutes of Health (NIH) (R01HS025164, R01HL144644). Dr Krumholz has received options for Element Science and Identifeye and payments from F-Prime for advisory roles; is a cofounder of and holds equity in Hugo Health, Refactor Health, and ENSIGHT-AI; and is associated with research contracts through Yale University from Janssen, Kenvue, Novartis, and Pfizer. Dr Yeh has received grant support from Abiomed, Astra Zeneca, and Boston Scientific, and consulting fees from Abbott, Boston Scientific, Medtronic, and Teleflex. Dr Curtis has a contract with the American College of Cardiology (ACC) for his role as Chief Scientific Advisor of the ACC’s National Cardiovascular Data Registry; and holds equity interest in Medtronic. Dr Higgins has reported that she has no relationships relevant to the contents of this paper to disclose. This research was supported by the American College of Cardiology’s National Cardiovascular Data Registry (NCDR). This research was also supported by the 10.13039/100000050National Heart, Lung, and Blood Institute (R01HL142765 and K23HL161424). The views expressed in this manuscript represent those of the author(s) and do not necessarily represent the official views of the NCDR or its associated professional societies identified at CVQuality.ACC.org/NCDR.
