# Supplementary material for: Comparing Claims Data to Stroke and Bleeding in the NCDR Left Atrial Appendage Occlusion Registry
Source: JACC Adv. 2025 Jul 24;4(8):102019. doi: 10.1016/j.jacadv.2025.102019 (PMC12311505; doi:10.1016/j.jacadv.2025.102019)
Supplement: Supplemental Material [file mmc1.pdf]

SUPPLEMENTAL APPENDIX

Supplemental Methods.

The current analysis compared adjudicated events in the LAAO registry to administrative claims data in Medicare. Adjudication was based on previously developed automated adjudication algorithms (AAAs) developed for the NCDR LAAO Registry.[1] The AAAs are currently used for site reporting of the specific LAAO Registry stroke and bleeding events listed below. In order to compare registry events to events in claims data, LAAO Registry event definitions were combined to create specific event types (see additional notes below).

Stroke events subject to adjudication are defined in the LAAO Registry as follows:

**Ischemic Stroke**	An ischemic stroke is an acute episode of focal or global neurological dysfunction caused by brain, spinal cord, or retinal vascular injury as a result of infarction of central nervous system tissue.
**Hemorrhagic Stroke**	An acute episode of focal or global cerebral or spinal dysfunction caused by intraparenchymal, intraventricular, or subarachnoid hemorrhage. Note: Subdural hematomas are intracranial hemorrhagic events and not strokes.
**Undetermined Stroke**	A stroke of undetermined origin is defined as an acute episode of focal or global neurological dysfunction caused by presumed brain, spinal cord, or retinal vascular injury as a result of hemorrhage or infarction but with insufficient information to allow categorization as ischemic or hemorrhagic.

Major bleeding events subject to adjudication are defined in the LAAO Registry as follows:

**Intracranial Hemorrhage (other than Hemorrhagic Stroke)***	Rapidly developing signs of neurologic dysfunction and/or headache due to bleeding into the subarachnoid space (the space between the arachnoid membrane and the pai mater of the brain and spinal cord). Subdural hematomas are also counted as intracranial hemorrhage.
**Major Bleeding (excluding Intracranial)****	Any bleeding requiring hospitalization, and/or causing a decrease in hemoglobin level ≥3g/dL, and/or requiring blood transfusion that was not hemorrhagic stroke.*

*In the current analysis comparing registry events to claims data, adjudicated “hemorrhagic stroke” and “intracranial hemorrhage other than hemorrhagic stroke” events were both counted as intracranial hemorrhage events.
**In the current analysis comparing registry events to claims data, adjudicated “hemorrhagic stroke”, “intracranial hemorrhage other than hemorrhagic stroke” and “major bleeding” (excluding intracranial) were counted as major bleeding events.

All stroke and bleeding events reported by the LAAO Registry in the current analysis were determined using specific criteria defined by the previously developed AAAs. Site-reported events that did not meet these specific criteria were not counted as registry events. The AAAs use multiple data elements from the LAAO Registry to determine whether a site-reported event is counted as a true clinical event. For stroke and intracranial hemorrhage events, LAAO Registry data on reported event type (ischemic stroke, hemorrhagic stroke, undetermined stroke, and intracranial hemorrhage other than hemorrhagic stroke) are combined with other reported variables on the LAAO Registry data collection form related to brain imaging, neurologic deficits, and assessment by a neurologist. In the current analysis, intracranial hemorrhage events in the LAAO Registry were defined as either a hemorrhagic stroke (identified by adjudication methods reported below) or intracranial hemorrhage (site-reported only; additional adjudication was not available for this specific outcome).

For major bleeding other than intracranial bleeding, reported bleeding type was combined with LAAO Registry data collection form variables on blood transfusion, necessity for an invasive intervention, and change in hemoglobin. In the currently analysis, major bleeding events were defined as either intracranial bleeding or major non-intracranial bleeding, as described by the adjudication methods reported below. GI bleeding was defined as adjudicated major bleeding with GI bleeding selected as the bleeding event type, and other bleeding was defined as adjudicated non-intracranial major bleeding for all other bleeding event types.

The detailed AAAs for endpoint adjudication using data elements on the LAAO Registry data collection form are shown below.

**ISCHEMIC STROKE**
**CONFIRMATION METHOD I**
Event Name = "Ischemic Stroke" or "Undetermined Stroke"
Brain Imaging Performed = "Yes"

Deficit Type = "Infarction" selected
**CONFIRMATION METHOD II**
Event Name = "Ischemic Stroke"
Brain Imaging Performed = "Yes"
Deficit Type = "Both" selected
Diagnosis confirmed by Neurology = "Yes"
**CONFIRMATION METHOD IIA**
Event Name = "Ischemic Stroke"
Brain Imaging Performed = "No"
Diagnosis confirmed by Neurology = "Yes"
**CONFIRMATION METHOD IIB**
Event Name = "Ischemic Stroke"
Brain Imaging Performed = "Yes"
Deficit Type = "No deficit" selected
Diagnosis confirmed by Neurology = "Yes"
**CONFIRMATION METHOD III**
Event Name = "TIA"
Brain Imaging Performed = "Yes"
Deficit Type = "Infarction" selected
Neurologic deficit with Rapid Onset = "Yes"
**HEMORRHAGIC STROKE***
**CONFIRMATION METHOD I**
Event Name = "Hemorrhagic Stroke " or "Undetermined Stroke"
Brain Imaging Performed = "Yes"
Deficit Type = "Hemorrhage" selected
**CONFIRMATION METHOD II**
Event Name = "Hemorrhagic Stroke "
Brain Imaging Performed = "Yes"
Deficit Type ="Both" selected
Diagnosis confirmed by Neurology = "Yes"
**CONFIRMATION METHOD III**
Event Name = "TIA"
Brain Imaging Performed = "Yes"
Deficit Type = "Hemorrhage"
Neurologic deficit with Rapid Onset = "Yes"
**CONFIRMATION METHOD IV**
Event Name = "Intracranial Hemorrhage (other than hemorrhagic stroke)"
Brain Imaging Performed = "Yes"
Deficit Type = "Hemorrhage" selected

Hemorrhage Type = "Intracerebral"
**UNDETERMINED STROKE**
**CONFIRMATION METHOD I**
Event Name = "Undetermined Stroke"
Diagnosis confirmed by Neurology = "Yes"
**CONFIRMATION METHOD II**
Event Name = "Undetermined Stroke"
Neurologic Deficit with Rapid Onset = "Yes"
Symptom Duration = "> 24 hrs"
Brain imaging performed = "No"
**CONFIRMATION METHOD III**
Event Name = "Undetermined Stroke"
If Deceased, Cause of Death = "Stroke"
No if confirmed as "Stroke, Ischemic" or "Stroke, Hemorrhagic"
**MAJOR BLEEDING (EXCLUDING INTRACRANIAL BLEEDING)****
**CONFIRMATION METHOD I**
Event Name = "Access Site Bleeding" OR "Hematoma" OR "GI Bleeding " OR "Retroperitoneal Bleeding" OR "Other Hemorrhage (non-intracranial)" OR "Vascular Complications"
Any transfusion OR Invasive Intervention OR Hemoglobin Drop >= 3 g/dl
**CONFIRMATION METHOD II**
If Deceased, Cause of Death = "Cardiovascular Hemorrhage" OR "Hemorrhage"

*In the LAAO Registry, hemorrhagic stroke is reported separately from other types of intracranial hemorrhage. In the current analysis comparing registry events to claims data, adjudicated "hemorrhagic stroke" and site-reported "intracranial hemorrhage other than hemorrhagic stroke" events were both counted as intracranial hemorrhage events.

**In the LAAO Registry, major bleeding is reported separately from intracranial hemorrhage. In the current analysis comparing registry events to claims data, adjudicated major bleeding events (excluding intracranial) and intracranial hemorrhage events (defined in the preceding footnote) were both counted as major bleeding events. GI bleeding events in the current analysis were defined as adjudicated major bleeding events (excluding intracranial) in which "GI Bleeding" was selected as the event type. Other bleeding events in the current analysis were defined as adjudicated major bleeding events (excluding intracranial) for all event types other than GI bleeding.

In certain scenarios, additional manual adjudication is performed on a mandatory basis to ensure accuracy for site-reported neurologic events and death in the LAAO Registry (criteria listed below). In the current analysis, only manually adjudicated events were used for registry events meeting these criteria.

**LAAO Registry Criteria for Mandatory Clinical Events Committee Adjudication after Application of the Automated Adjudication Algorithm**
**NEUROLOGIC EVENTS**
If the AAA algorithm adjudicates two or more neurologic events (ischemic stroke, hemorrhagic stroke, undetermined stroke, TIA or intracranial hemorrhage) in the same events

If the AAA algorithm determines there are two neurologic events (ischemic stroke, hemorrhagic stroke, undetermined stroke, TIA or intracranial hemorrhage) in the same patient within one month
If a site reports a neurologic event (ischemic stroke, hemorrhagic stroke, undetermined stroke, TIA or intracranial hemorrhage) and the AAA reports no neurologic event or a different type of neurologic event
If a site reports a neurologic event (ischemic stroke, hemorrhagic stroke, undetermined stroke, TIA or intracranial hemorrhage) and no brain imaging is performed
If a site reports a neurologic event (ischemic stroke, hemorrhagic stroke, undetermined stroke, TIA or intracranial hemorrhage) and brain imaging is performed but there is no deficit reported on imaging
**DEATH**
If a site reports death and the cause of death is listed as hemorrhage, cardiovascular hemorrhage or stroke

References:

1. Friedman DJ, Pierre D, Wang Y, Gambone L, Koutras C, Segawa C, Farb A, Vemulapalli S, Varosy PD, Masoudi FA, Lansky A, Curtis JP and Freeman JV. Development and validation of an automated algorithm for end point adjudication for a large U.S. national registry. *Am Heart J*. 2022;254:102-111.

Supplemental Table 1. ICD-10 Codes for Stroke Events

Ischemic Stroke	ICD-10 codes	Description
	**I63.***	**Cerebral Infarction**
	**I97.810, I97.811, I97.820, I97.821**	**Intraoperative or Postprocedural Cerebrovascular Infarction**
**Hemorrhagic Stroke**	**ICD-10 codes**	**Description**
	**I60.***	**Nontraumatic subarachnoid hemorrhage**
	**I61.***	**Nontraumatic intracerebral hemorrhage**

*Includes all subcodes.

Supplemental Table 2. ICD-10 Codes for Bleeding Events

Intracranial Bleeding	ICD-10 codes	Description
	I60.*	Nontraumatic subarachnoid hemorrhage
	I61.*	Nontraumatic intracerebral hemorrhage
	I62.*	Other and unspecified nontraumatic intracranial hemorrhage
	S06.34, S06.34*, S06.35, S06.35*, S06.36, S06.36*	Traumatic hemorrhage of cerebrum
	S06.37, S06.37*	Contusion, laceration and hemorrhage of cerebellum
	S06.38, S06.38*	Contusion, laceration and hemorrhage of brain stem
	S06.4, S06.4X*	Epidural hemorrhage
	S06.5, S06.5X*	Traumatic subdural hemorrhage
	S06.6, S06.6X*	Traumatic subarachnoid hemorrhage
**GI Bleeding**	**ICD-10 codes**	**Description**
	I85.01, I85.11	Esophageal varices with bleeding
	K21.01	Gastro-esophageal reflux disease with esophagitis, with bleeding
	K22.11, K22.81	Ulcer of esophagus with bleeding, esophageal hemorrhage
	K22.6	Gastro-esophageal laceration-hemorrhage syndrome (Mallory-Weiss syndrome)
	K25.0, K25.2, K25.4, K25.6	Gastric ulcer (acute or chronic) with hemorrhage (with or without perforation)
	K26.0, K26.2, K26.4, K26.6	Duodenal ulcer (acute or chronic) with hemorrhage (with or without perforation)
	K27.0, K27.2, K27.4, K27.6	Peptic ulcer (acute or chronic) with hemorrhage (with or without perforation)
	K28.0, K28.2, K28.4, K28.6	Gastrojejunal ulcer (acute or chronic) with hemorrhage (with or without perforation)
	K29.*1	Gastritis and duodenitis with bleeding
	K31.811, K55.21	Angiodysplasia of stomach, duodenum, or colon with hemorrhage
	K31.82, K63.81	Dieulafoy lesion of stomach, duodenum, or intestine
	K50.011, K50.111, K50.811, K50.911	Crohn's disease with rectal bleeding
	K51.011, K51.211, K51.311, K51.411, K51.511, K51.811, K51.911	Ulcerative colitis with rectal bleeding
	K57.01, K57.11, K57.13, K57.21, K57.31, K57.33, K57.41, K57.51, K57.53, K57.81, K57.91, K57.93	Diverticulosis or diverticulitis of small or large intestine with bleeding
	K62.5	Hemorrhage of anus and rectum
	K64.0, K64.1, K64.2, K64.3, K64.8, K64.9	Hemorrhoids
	K92.0, K92.1, K92.2	Gastrointestinal hemorrhage or melena
	K94.01, K94.11, K94.21, K94.31	Colostomy or enterostomy or esophagostomy or gastrostomy hemorrhage
**Other Bleeding**	**ICD-10 codes**	**Description**
	D50.0	Iron deficiency anemia secondary to blood loss (chronic)
	D62	Acute posthemorrhagic anemia
	D68.32	Hemorrhagic disorder due to extrinsic circulating anticoagulants
	H05.231, H05.232, H05.233, H05.239	Hemorrhage of orbit
	H11.3*	Conjunctival hemorrhage

	**H31.3***	**Choroidal hemorrhage**
	**H35.6***	**Retinal hemorrhage**
	**H43.1***	**Vitreous hemorrhage**
	**H61.12***	**Hematoma of pinna**
	**I31.2**	**Hemopericardium, not elsewhere classified**
	**I77.2**	**Rupture of artery**
	**J94.2**	**Hemothorax**
	**K66.1**	**Hemoperitoneum**
	**K76.2**	**Central hemorrhagic necrosis of liver**
	**M25.0, M25.0***	**Hemarthrosis**
	**M79.81**	**Nontraumatic hematoma of soft tissue**
	**N02, N02.*, N30.01, N30.91, R31, R31.***	**Hematuria**
	**N32.89**	**Hemorrhage into bladder wall**
	**N42.1**	**Congestion and hemorrhage of prostate**
	**N92.4**	**Excessive bleeding in the premenopausal period**
	**N93.8**	**Other specified abnormal uterine and vaginal bleeding**
	**N93.9**	**Abnormal uterine and vaginal bleeding, unspecified**
	**N95**	**Postmenopausal bleeding**
	**R04.0**	**Epistaxis**
	**R04.1**	**Hemorrhage from throat**
	**R04.2**	**Hemoptysis**
	**R04.8***	**Hemorrhage from other sites in respiratory passages**
	**R04.9**	**Hemorrhage from respiratory passages, unspecified**
	**R23.3**	**Spontaneous ecchymoses**
	**R58**	**Hemorrhage, not elsewhere classified**
	**S00.03*, S00.1*, S00.33*, S00.43*, S00.53.*, S00.83*, S00.93*, S05.1*, S10.0*, S10.83*, S20.0*, S20.2*, S26.0*, S26.1*, S26.91*, S27.32*, S27.42*, S27.52*, S27.802*, S27.812*, S27.892*, S30.0*, S30.1*, S30.2*, S30.3*, S36.02*, S36.112*, S36.122*, S36.22*, S36.32*, S36.42*, S36.52*, S36.62*, S36.892*, S36.92*, S37.01*, S37.02*, S37.12*, S37.22*, S37.32*, S37.42*, S37.52*, S37.62*, S37.812*, S37.822*, S37.892*, S37.92*, S40.0*, S50.0*, S50.1*, S60.0*, S60.1*, S60.2*, S70.0*, S70.1*, S80.0*, S80.1*, S90.0*, S90.1*, S90.2*, S90.3***	**Contusion (other than intracranial)**
	**T82.83*, T83.83*, T84.83*, T85.83***	**Hemorrhage due to prosthetic devices, implants and grafts**

	**D78.0*, D78.22, D78.32, E36.02, E89.82*, G97.3*, G97.5*, G97.61, G97.62, H59.1*, H59.31*, H59.32*, H59.33*, H59.34*, H95.2*, H95.4*, H95.5*, I97.4*, I97.61*, I97.62*, I97.63*, J95.01, J95.6*, J95.83*, J95.860, J95.861, K91.61, K91.62, K91.840, K91.841, K91.870, K91.871, L76.0*, L76.2*, L76.31, L76.32, M96.81*, M96.83*, M96.840, M96.841, N99.510, N99.520, N99.530, N99.6*, N99.82*, N99.840, N99.841**	**Intra-Procedural and Post-Procedural Bleeding**
**Red Blood Cell Transfusion**		
	**30230H0, 30230H1, 30230N0, 30230N1, 30230P0, 30230P1, 30233H0, 30233H1, 30233N0, 30233N1, 30233P0, 30233P1, 30240H0, 30240H1, 30240N0, 30240N1, 30240P0, 30240P1, 30243H0, 30243H1, 30243N0, 30243N1, 30243P0, 30243P1, 30250H0, 30250H1, 30250N0, 30250N1, 30250P0, 30250P1, 30253H0, 30253H1, 30253N0, 30253N1, 30253P0, 30253P1, 30260H0, 30260H1, 30260N0, 30260N1, 30260P0, 30260P1, 30263H0, 30263H1, 30263N0, 30263N1, 30263P0, 30263P1**	**ICD-10-PCS codes for red blood cell transfusion**

*Includes all subcodes.

Supplemental Table 3. Positive Predictive Values of ICD-10 Codes Used for Diagnosis of Stroke and Bleeding Events

Outcome	ICD-10 code	Description	No. of Claims Events Matched with Registry Event	Total No. of Events with ICD-10 code(s)	Positive Predictive Value (%)
**Ischemic Stroke**	I63.*	Cerebral Infarction	602	1192	50.5
	I97.810, I97.811, I97.820, I97.821	Postprocedural Cerebrovascular Infarction	5	9	55.6
**Hemorrhagic Stroke**	I60.*	Nontraumatic subarachnoid hemorrhage	10	32	31.3
	I61.*	Nontraumatic intracerebral hemorrhage	93	172	54.1
**Intracranial Bleeding**	I60.*	Nontraumatic subarachnoid hemorrhage	14	32	43.8
	I61.*	Nontraumatic intracerebral hemorrhage	100	170	58.8
	I62.*	Other and unspecified nontraumatic intracranial hemorrhage	28	68	41.2
	S06.34, S06.34*, S06.35, S06.35*, S06.36, S06.36*	Traumatic hemorrhage of cerebrum	7	39	17.9
	S06.37, S06.37*	Contusion, laceration and hemorrhage of cerebellum	2	6	33.3
	S06.38, S06.38*	Contusion, laceration and hemorrhage of brain stem	0	0	-
	S06.4, S06.4X*	Epidural hemorrhage	0	4	0.0
	S06.5, S06.5X*	Traumatic subdural hemorrhage	122	380	32.1
	S06.6, S06.6X*	Traumatic subarachnoid hemorrhage	72	208	34.6
**Gastrointestinal Bleeding**	I85.01, I85.11	Esophageal varices with bleeding	4	10	40.0
	K21.01	Gastro-esophageal reflux disease with esophagitis, with bleeding	2	8	25.0
	K22.11, K22.81	Ulcer of esophagus with bleeding, esophageal hemorrhage	20	51	39.2
	K22.6	Gastro-esophageal laceration-hemorrhage syndrome (Mallory-Weiss syndrome)	9	19	47.4
	K25.0, K25.2, K25.4, K25.6	Gastric ulcer (acute or chronic) with hemorrhage (with or without perforation)	151	332	45.5
	K26.0, K26.2, K26.4, K26.6	Duodenal ulcer (acute or chronic) with hemorrhage (with or without perforation)	72	151	47.7
	K27.0, K27.2, K27.4, K27.6	Peptic ulcer (acute or chronic) with hemorrhage (with or without perforation)	7	11	63.6
	K28.0, K28.2, K28.4, K28.6	Gastrojejunal ulcer (acute or chronic) with hemorrhage (with or without perforation)	31	57	54.4
	K29.*1	Gastritis and duodenitis with bleeding	97	262	37.0

	K31.811, K55.21	Angiodysplasia of stomach, duodenum, or colon with hemorrhage	591	1201	49.2
	K31.82, K63.81	Dieulafoy lesion of stomach, duodenum, or intestine	46	82	56.1
	K50.011, K50.111, K50.811, K50.911	Crohn's disease with rectal bleeding	5	20	25.0
	K51.011, K51.211, K51.311, K51.411, K51.511, K51.811, K51.911	Ulcerative colitis with rectal bleeding	5	24	20.8
	K57.*1, K57.*3, K57.01, K57.11, K57.13, K57.21, K57.31, K57.33, K57.41, K57.51, K57.53, K57.81, K57.91, K57.93	Diverticulosis or diverticulitis of small or large intestine with bleeding	329	917	35.9
	K62.5	Hemorrhage of anus and rectum	20	101	19.8
	K64.0, K64.1, K64.2, K64.3, K64.8, K64.9	Hemorrhoids	36	136	26.4
	K92.0, K92.1, K92.2	Gastrointestinal hemorrhage or melena	662	1793	36.9
	K94.01, K94.11, K94.21, K94.31	Colostomy or enterostomy or esophagostomy or gastrostomy hemorrhage	2	13	15.4
**Other Bleeding**	D50.0	Iron deficiency anemia secondary to blood loss (chronic)	11	191	5.8
	D62	Acute posthemorrhagic anemia	35	530	6.6
	D68.32	Hemorrhagic disorder due to extrinsic circulating anticoagulants	10	276	3.6
	H05.231, H05.232, H05.233, H05.239	Hemorrhage of orbit	0	0	-
	H11.3*	Conjunctival hemorrhage	0	1	0.0
	H31.3*	Choroidal hemorrhage	0	0	-
	H35.6*	Retinal hemorrhage	0	1	0.0
	H43.1*	Vitreous hemorrhage	0	1	0.0
	H61.12*	Hematoma of pinna	0	0	-
	I31.2	Hemopericardium, not elsewhere classified	2	6	33.3
	I77.2	Rupture of artery	0	1	0.0
	J94.2	Hemothorax	3	13	23.1
	K66.1	Hemoperitoneum	4	18	22.2
	K76.2	Central hemorrhagic necrosis of liver	0	0	-
	M25.0, M25.0*	Hemarthrosis	1	12	8.3
	M79.81	Nontraumatic hematoma of soft tissue	6	34	17.6
	N02, N02.*, N30.01, N30.91, R31, R31.*	Hematuria	17	225	7.6
	N32.89	Hemorrhage into bladder wall	2	12	16.7

	N42.1	Congestion and hemorrhage of prostate	2	6	33.3
	N92.4	Excessive bleeding in the premenopausal period	0	0	-
	N93.8	Other specified abnormal uterine and vaginal bleeding	0	1	0.0
	N93.9	Abnormal uterine and vaginal bleeding, unspecified	0	3	0.0
	N95	Postmenopausal bleeding	0	3	0.0
	R04.0	Epistaxis	18	85	21.2
	R04.1	Hemorrhage from throat	0	1	0.0
	R04.2	Hemoptysis	3	22	13.6
	R04.8*	Hemorrhage from other sites in respiratory passages	1	3	33.3
	R04.9	Hemorrhage from respiratory passages, unspecified	0	0	-
	R23.3	Spontaneous ecchymoses	1	1	100.0
	R58	Hemorrhage, not elsewhere classified	1	2	50.0
	S00.03*, S00.1*, S00.33*, S00.43*, S00.53.*, S00.83*, S00.93*, S05.1*, S10.0*, S10.83*, S20.0*, S20.2*, S26.0*, S26.1*, S26.91*, S27.32*, S27.42*, S27.52*, S27.802*, S27.812*, S27.892*, S30.0*, S30.1*, S30.2*, S30.3*, S36.02*, S36.112*, S36.122*, S36.22*, S36.32*, S36.42*, S36.52*, S36.62*, S36.892*, S36.92*, S37.01*, S37.02*, S37.12*, S37.22*, S37.32*, S37.42*, S37.52*, S37.62*, S37.812*, S37.822*, S37.892*, S37.92*, S40.0*, S50.0*, S50.1*, S60.0*, S60.1*, S60.2*, S70.0*, S70.1*, S80.0*, S80.1*, S90.0*, S90.1*, S90.2*, S90.3*	Contusion (other than intracranial)	22	198	11.1
	T82.83*, T83.83*, T84.83*, T85.83*	Hemorrhage due to prosthetic devices, implants and grafts	8	48	16.7

	D78.0*, D78.22, D78.32, E36.02, E89.82*, G97.3*, G97.5*, G97.61, G97.62, H59.1*, H59.31*, H59.32*, H59.33*, H59.34*, H95.2*, H95.4*, H95.5*, I97.4*, I97.61*, I97.620, I97.621, I97.63*, J95.01, J95.6*, J95.83*, J95.860, J95.861, K91.61, K91.62, K91.840, K91.841, K91.870, K91.871, L76.0*, L76.2*, L76.31, L76.32, M96.81*, M96.83*, M96.840, M96.841, N99.510, N99.520, N99.530, N99.6*, N99.82*, N99.840, N99.841	Intra-Procedural and Post-Procedural Bleeding	50	280	17.9

Supplemental Table 4. Other Non-Event ICD-10 Codes Used for the Primary Diagnosis of Hospitalizations Occurring at the Time of a Registry-Reported Event*

Outcome	ICD-10 code	Description	Total No. of Events with ICD-10 code
**Ischemic Stroke**	A41	Other sepsis	16
	G45	Transient cerebral ischemic attacks and related syndromes	15
	I48	Atrial fibrillation and flutter	31
	I69	Sequelae of cerebrovascular disease	30
	S06	Intracranial injury	11
	T82	Complications of cardiac and vascular prosthetic devices, implants and grafts	17
**Hemorrhagic Stroke**	I62	Other and unspecified nontraumatic intracranial hemorrhage	12
	I63	Cerebral infarction	20
	I69	Sequelae of cerebrovascular disease	10
	S06	Intracranial injury	32
**Intracranial Bleeding**	A41	Other sepsis	10
	I48	Atrial fibrillation and flutter	18
	I63	Cerebral infarction	29
	I69	Sequelae of cerebrovascular disease	13
**Gastrointestinal Bleeding**	A41	Other sepsis	70
	C18	Malignant neoplasm of colon	20
	D50	Iron deficiency anemia	83
	D62	Acute posthemorrhagic anemia	179
	D64	Other anemias	33
	D68	Other coagulation defects	99
	I11	Hypertensive heart disease	23
	I13	Hypertensive heart and chronic kidney disease	75
	I48	Atrial fibrillation and flutter	233
	I50	Heart failure	12
	J15	Bacterial pneumonia, not elsewhere classified	11
	J18	Pneumonia, unspecified organism	12
	J96	Respiratory failure, not elsewhere classified	11
	K22 (excluding K22.6)	Other diseases of esophagus	10
	K25 (excluding K25.0, K25.2, K25.4 and K25.6)	Gastric ulcer	10
	K31 (excluding K31.811, K31.82)	Other diseases of stomach and duodenum	28
	K55	Vascular disorders of intestine	36
	K57 (excluding codes with bleeding; see Supp. Table 4)	Diverticular disease of intestine (excluding with bleeding)	10
	K62 (excluding K62.5)	Other diseases of anus and rectum	36

	K63	Other diseases of intestine	12
	K74	Fibrosis and cirrhosis of liver	12
	K76	Other diseases of liver	12
	K91	Intraoperative and postprocedural complications and disorders of digestive system, not elsewhere classified	26
	K92 (excluding K92.0, K92.1, K92.2)	Other diseases of digestive system	17
	N17	Acute kidney failure	19
	Q27	Other congenital malformations of peripheral vascular system	15
	T82	Complications of cardiac and vascular prosthetic devices, implants and grafts	10
	U07.1	COVID-19	12
**Other Bleeding**	A41	Other sepsis	33
	D50.9	Iron deficiency anemia, unspecified	11
	D64	Other anemias	10
	I11	Hypertensive heart disease	16
	I13	Hypertensive heart and chronic kidney disease	44
	I21	Acute myocardial infarction	11
	I30	Acute pericarditis	10
	I31.3	Pericardial effusion (noninflammatory)	74
	I48	Atrial fibrillation and flutter	159
	I72	Other aneurysm	20
	I97.89	Other postprocedural complications and disorders of circulatory system, not elsewhere classified	10
	J90	Pleural effusion, not elsewhere classified	13
	J96	Respiratory failure, not elsewhere classified	13
	K92	Other diseases of digestive system	17
	N17	Acute kidney failure	17
	S06	Intracranial injury	12
	S72	Fracture of femur	19
	T81	Complications of procedures, not elsewhere classified	13
	T82 (excluding T82.83)	Complications of cardiac and vascular prosthetic devices, implants and grafts	25

*****Other non-event ICD-10 codes reported as the primary diagnosis for hospitalizations in claims data occurring on the date of a reported event in the LAAO Registry ± 14 days (i.e. “false negatives,” using the registry as the reference) are shown. For individual events, hospitalizations in which the primary diagnosis code was defined as an event (as per Supplemental Tables 1 & 2) and matched to the specific registry event (i.e. “true positives”) were therefore not included. Only ICD-10 codes with ≥10 events for each outcome are shown.

Supplemental Table 5. Stroke Events in Claims Data Compared to Registry Data with Different Claims-Based Event Definitions

Clinical Event Definition in Claims Data	No. of Events in Registry*	No. of Events in Claims*	Sensitivity,% (95% CI)	Specificity,% (95% CI)	PPV,% (95% CI)	NPV,% (95% CI)	Kappa Coefficient (95% CI)
**All Stroke Events**							
Primary Codes ± 14 days (primary analysis)	1308	1405	58.1 (55.4, 60.8)	99.1 (99.0, 99.2)	54.1 (51.9, 56.3)	99.2 (99.2, 99.3)	0.55 (0.53-0.57)
Primary Codes ± 7 days	1308	1405	57.6 (54.9, 60.3)	99.07 (99.0, 99.1)	53.6 (51.37, 55.82)	99.2 (99.2, 99.3)	0.55 (0.52-0.57)
Primary Codes ± 30 days	1308	1405	58.9 (56.3, 61.6)	99.1 (99.0, 99.1)	54.8 (52.6, 57.0)	99.2 (99.2, 99.3)	0.56 (0.54-0.58)
Primary Codes ± 14 days – Excluding Patients with Prior Stroke	713	764	56.0 (52.3, 59.6)	99.3 (99.3, 99.4)	52.2 (49.2, 55.3)	99.4 (99.4, 99.5)	0.53 (0.50-0.57)
Primary/Secondary Codes ± 7 days	1308	1831	66.3 (63.7, 68.9)	98.6 (98.5, 98.7)	47.4 (45.5, 49.2)	99.4 (99.3, 99.4)	0.54 (0.52-0.56)
Primary/Secondary Codes ± 14 days	1308	1833	67.0 (64.4, 69.5)	98.6 (98.6, 98.7)	47.8 (46.0, 49.6)	99.4 (99.3, 99.4)	0.55 (0.53-0.57)
Primary/Secondary Codes ± 30 days	1308	1831	68.0 (65.4, 70.5)	98.7 (98.6, 98.7)	48.6 (46.7, 50.4)	99.4 (99.4, 99.4)	0.56 (0.54-0.58)
Primary/Secondary Codes ± 14 days – Excluding Patients with Prior Stroke	713	1006	65.8 (62.3, 69.3)	99.0 (98.9, 99.1)	46.6 (44.2, 49.1)	99.6 (99.5, 99.6)	0.54 (0.51-0.57)
**Ischemic Stroke Events**							
Primary Codes ± 14 days (primary analysis)	998	1201	60.8 (57.8, 63.9)	99.2 (99.1, 99.2)	50.5 (48.2, 52.9)	99.4 (99.4, 99.5)	0.55 (0.52-0.57)
Primary Codes ± 7 days	998	1201	60.2 (57.2, 63.3)	99.2 (99.1, 99.2)	50.0 (47.7, 52.4)	99.4 (99.4, 99.5)	0.54 (0.51-0.56)
Primary Codes ± 30 days	998	1201	61.5 (58.5, 64.5)	99.2 (99.1, 99.3)	51.1 (48.8, 53.5)	99.5 (99.4, 99.5)	0.55 (0.53-0.58)
Primary Codes ± 14 days – Excluding Patients with Prior Stroke	539	648	58.8 (54.7, 63.0)	99.4 (99.3, 99.5)	48.9 (45.7, 52.1)	99.6 (99.6, 99.6)	0.53 (0.49-0.56)
Primary/Secondary Codes ± 7 days	998	1567	69.5 (66.7, 72.4)	98.8 (98.7, 98.8)	44.3 (42.4, 46.2)	99.6 (99.5, 99.6)	0.53 (0.51-0.56)
Primary/Secondary Codes ± 14 days	998	1567	70.3 (67.5, 73.2)	98.8 (98.7, 98.9)	44.8 (42.9, 46.7)	99.6 (99.5, 99.6)	0.54 (0.52-0.56)
Primary/Secondary Codes ± 30 days	998	1567	71.3 (68.5, 74.2)	98.8 (98.7, 98.9)	45.4 (43.5, 47.4)	99.6 (99.6, 99.6)	0.55 (0.52-0.57)
Primary/Secondary Codes ± 14 days – Excluding Patients with Prior Stroke	539	851	69.8 (65.9, 73.6)	99.1 (99.1, 99.2)	44.2 (41.6, 46.8)	99.7 (99.7, 99.7)	0.54 (0.50-0.57)
**Hemorrhagic Stroke Events**							
Primary Codes ± 14 days (primary analysis)	241	204	42.7 (36.5, 49.0)	99.9 (99.8, 99.9)	50.5 (44.4, 56.6)	99.8 (99.8, 99.8)	0.46 (0.40-0.52)
Primary Codes ± 7 days	241	204	42.7	99.9	50.5	99.8	0.46 (0.40-0.52)

			(36.5, 49.0)	(99.8, 99.9)	(44.4, 56.6)	(99.8, 99.8)	
Primary Codes ± 30 days	241	204	43.6 (37.3, 49.8)	99.9 (99.8, 99.9)	51.5 (45.4, 57.6)	99.8 (99.8, 99.8)	0.47 (0.41-0.53)
Primary Codes ± 14 days – Excluding Patients with Prior Stroke	136	116	44.1 (35.8, 52.5)	99.9 (99.9, 99.9)	51.7 (43.7, 59.8)	99.9 (99.8, 99.9)	0.48 (0.40-0.55)
Primary/Secondary Codes ± 7 days	241	374	53.9 (47.7, 60.2)	99.7 (99.6, 99.7)	34.8 (30.9, 38.6)	99.8 (99.8, 99.9)	0.42 (0.37-0.47)
Primary/Secondary Codes ± 14 days	241	374	53.9 (47.7, 60.2)	99.7 (99.6, 99.7)	34.8 (30.9, 38.6)	99.8 (99.8, 99.9)	0.42 (0.37-0.47)
Primary/Secondary Codes ± 30 days	241	373	54.4 (48.1, 60.7)	99.7 (99.6, 99.7)	35.1 (31.2, 39.0)	99.9 (99.8, 99.9)	0.42 (0.38-0.47)
Primary/Secondary Codes ± 14 days – Excluding Patients with Prior Stroke	136	209	54.4 (46.0, 62.8)	99.8 (99.7, 99.8)	35.4 (30.2, 40.6)	99.9 (99.9, 99.9)	0.43 (0.36-0.49)

*The total number of events in the LAAO Registry and in claims data can vary with similar definitions due to differences in how overlapping events are accounted for (see Methods).

Supplemental Table 6. Clinical Events in Claims Data Compared to Registry Data for Men and Women

Clinical Event Definition in Claims Data	No. of Events in Registry*	No. of Events in Claims*	Sensitivity,% (95% CI)	Specificity,% (95% CI)	PPV,% (95% CI)	NPV,% (95% CI)	Kappa Coefficient (95% CI)
**All Stroke Events**							
Primary Codes ± 14 days – Men	722	807	57.9 (54.3, 61.5)	99.1 (99.0, 99.1)	51.8 (48.9, 54.7)	99.3 (99.2, 99.32)	0.54 (0.51-0.57)
Primary Codes ± 14 days – Women	586	597	58.4 (54.4, 62.4)	99.1 (99.0, 99.2)	57.3 (53.9, 60.7)	99.2 (99.1, 99.2)	0.57 (0.54-0.60)
Primary/Secondary Codes ± 14 days – Men	722	1057	66.9 (63.5, 70.3)	98.6 (98.5, 98.7)	45.7 (43.3, 48.1)	99.4 (99.4, 99.5)	0.53 (0.51-0.56)
Primary/Secondary Codes ± 14 days – Women	586	775	67.1 (63.3, 70.9)	98.7 (98.6, 98.8)	50.7 (47.8, 53.6)	99.3 (99.3, 99.4)	0.57 (0.54-0.60)
**Ischemic Stroke Events**							
Primary Codes ± 14 days – Men	540	688	60.9 (56.8, 65.0)	99.1 (99.0, 99.2)	47.8 (44.8, 50.9)	99.5 (99.4, 99.5)	0.53 (0.49-0.56)
Primary Codes ± 14 days – Women	458	512	60.7 (56.2, 65.2)	99.2 (99.1, 99.3)	54.3 (50.6, 58.0)	99.4 (99.3, 99.5)	0.57 (0.53-0.60)
Primary/Secondary Codes ± 14 days – Men	540	900	70.7 (66.9, 74.6)	98.7 (98.6, 98.8)	42.4 (40.0, 44.9)	99.6 (99.6, 99.7)	0.52 (0.49-0.55)
Primary/Secondary Codes ± 14 days – Women	458	666	69.9 (65.7, 74.1)	98.8 (98.7, 98.9)	48.1 (45.0, 51.1)	99.5 (99.5, 99.5)	0.56 (0.53-0.60)
**Hemorrhagic Stroke Events**							
Primary Codes ± 14 days – Men	145	119	42.8 (34.7, 50.8)	99.9 (99.8, 99.9)	52.1 (44.1, 60.1)	99.8 (99.8, 99.8)	0.47 (0.39-0.54)
Primary Codes ± 14 days – Women	96	85	42.7 (32.8, 52.6)	99.9 (99.8, 99.9)	48.2 (38.9, 57.6)	99.8 (99.8, 99.9)	0.45 (0.36-0.54)
Primary/Secondary Codes ± 14 days – Men	145	213	55.2 (47.1, 63.3)	99.7 (99.6, 99.7)	37.6 (32.3, 42.8)	99.8 (99.8, 99.9)	0.44 (0.38-0.51)
Primary/Secondary Codes ± 14 days – Women	96	160	52.1 (42.1, 62.1)	99.6 (99.6, 99.7)	31.3 (25.5, 37.0)	99.8 (99.8, 99.9)	0.38 (0.31-0.46)
**All Major Bleeding Events**							
Primary Codes ± 14 days – Men	2816	4408	57.1 (55.3, 59.0)	93.0 (92.8, 93.3)	36.5 (35.4, 37.6)	96.9 (96.7, 97.0)	0.40 (0.38-0.41)
Primary Codes ± 14 days – Women	2435	3648	57.5 (55.5, 59.5)	92.1 (91.8, 92.4)	38.4 (37.1, 39.6)	96.2 (96.0, 96.4)	0.40 (0.39-0.42)
Primary/Secondary Codes ± 14 days – Men	2809	9699	72.5 (70.8, 74.1)	81.7 (81.3, 82.1)	21.0 (20.5, 21.5)	97.8 (97.7, 97.9)	0.25 (0.24-0.26)
Primary/Secondary Codes ± 14 days – Women	2433	7701	72.6 (70.8, 74.4)	80.2 (79.8, 80.7)	22.9 (22.3, 23.5)	97.3 (97.1, 97.5)	0.26 (0.25-0.28)
**Intracranial Hemorrhage Events**							

Primary Codes ± 14 days – Men	314	565	63.1 (57.7, 68.4)	99.1 (99.0, 99.2)	35.0 (32.0, 38.1)	99.7 (99.7, 99.8)	0.45 (0.40-0.49)
Primary Codes ± 14 days – Women	241	370	61.0 (54.8, 67.2)	99.2 (99.2, 99.3)	39.7 (35.8, 43.7)	99.7 (99.6, 99.7)	0.48 (0.43-0.53)
Primary/Secondary Codes ± 14 days – Men	314	756	72.6 (67.7, 77.5)	98.7 (98.6, 98.8)	30.2 (27.9, 32.5)	99.8 (99.8, 99.8)	0.42 (0.38-0.46)
Primary/Secondary Codes ± 14 days – Women	241	517	72.6 (67.0, 78.2)	98.8 (98.7, 99.0)	33.9 (30.9, 36.8)	99.8 (99.7, 99.8)	0.46 (0.41-0.50)
**Gastrointestinal Bleeding Events**							
Primary Codes ± 14 days – Men	1961	2759	56.5 (54.3, 58.7)	95.9 (95.7, 96.1)	40.1 (38.7, 41.6)	97.8 (97.7, 98.0)	0.44 (0.42-0.46)
Primary Codes ± 14 days – Women	1778	2427	55.2 (52.9, 57.5)	95.0 (94.7, 95.2)	40.5 (38.9, 42.0)	97.2 (97.0, 97.3)	0.43 (0.41-0.45)
Primary/Secondary Codes ± 14 days – Men	1962	4511	73.3 (71.3, 75.3)	92.5 (92.2, 92.7)	31.9 (30.9, 32.8)	98.6 (98.5, 98.7)	0.41 (0.39-0.42)
Primary/Secondary Codes ± 14 days – Women	1781	4339	72.0 (69.9, 74.1)	89.7 (89.3, 90.0)	29.6 (28.6, 30.5)	98.2 (98.0, 98.3)	0.37 (0.35-0.38)
**Other Major Bleeding Events**							
Primary Codes ± 14 days – Men	545	1119	19.5 (16.1, 22.8)	97.5 (97.4, 97.7)	9.5 (7.9, 11.0)	98.9 (98.9, 99.0)	0.11 (0.09-0.13)
Primary Codes ± 14 days – Women	422	855	21.6 (17.6, 25.5)	97.4 (97.2, 97.6)	10.6 (8.8, 12.5)	98.9 (98.8, 98.9)	0.13 (0.10-0.15)
Primary/Secondary Codes ± 14 days – Men	539	7788	49.9 (45.7, 54.1)	82.6 (82.2, 82.9)	3.5 (3.2, 3.7)	99.3 (99.2, 99.3)	0.04 (0.04-0.05)
Primary/Secondary Codes ± 14 days – Women	421	6166	50.6 (45.8, 55.4)	80.9 (80.4, 81.3)	3.5 (3.1, 3.8)	99.2 (99.1, 99.3)	0.04 (0.03-0.05)

*The total number of events in the LAAO Registry and in claims data can vary with similar definitions due to differences in how overlapping events are accounted for (see Methods).

Supplemental Table 7. Bleeding Events in Claims Data Compared to Registry Data with Different Claims-Based Event Definitions

Clinical Event Definition in Claims Data	No. of Events in Registry*	No. of Events in Claims*	Sensitivity,% (95% CI)	Specificity,% (95% CI)	PPV,% (95% CI)	NPV,% (95% CI)	Kappa Coefficient (95% CI)
**All Major Bleeding Events**							
Primary Codes ± 14 days (primary analysis)	5253	8056	57.3 (56.0, 58.6)	92.6 (92.4, 92.8)	37.4 (36.5, 38.2)	96.6 (96.5, 96.7)	0.40 (0.39-0.41)
Primary Codes ± 7 days	5259	8068	56.5 (55.2, 57.9)	92.6 (92.4, 92.8)	36.9 (36.0, 37.7)	96.5 (96.4, 96.6)	0.39 (0.38-0.40)
Primary Codes ± 30 days	5231	8049	58.4 (57.1, 59.7)	92.7 (92.5, 92.9)	37.9 (37.1, 38.8)	96.7 (96.6, 96.8)	0.41 (0.40-0.42)
Primary Codes ± 14 days + RBC Transfusion*	5249	11851	64.2 (62.9, 65.5)	88.0 (87.7, 88.2)	28.4 (27.8, 29.0)	97.1 (97.0, 97.2)	0.33 (0.32-0.34)
Primary/Secondary Codes ± 14 days	5242	17406	72.5 (71.3, 73.7)	81.1 (80.8, 81.4)	21.8 (21.5, 22.2)	97.6 (97.5, 97.7)	0.26 (0.25-0.27)
Primary/Secondary Codes ± 7 days	5251	17431	71.5 (70.3, 72.7)	81.0 (80.7, 81.3)	21.5 (21.2, 21.9)	97.5 (97.4, 97.6)	0.25 (0.25-0.26)
Primary/Secondary Codes ± 30 days	5227	17387	73.9 (72.8, 75.1)	81.2 (80.9, 81.5)	22.2 (21.9, 22.6)	97.7 (97.6, 97.8)	0.27 (0.26-0.27)
Primary/Secondary Codes ± 14 days + RBC Transfusion**	5240	19610	74.0 (72.8, 75.2)	78.6 (78.3, 78.9)	19.8 (19.4, 20.1)	97.7 (97.6, 97.8)	0.23 (0.22-0.24)
**Intracranial Hemorrhage Events**							
Primary Codes ± 14 days (primary analysis)	555	907	62.2 (58.1, 66.2)	99.2 (99.1, 99.3)	38.0 (35.6, 40.5)	99.7 (99.7, 99.7)	0.47 (0.44-0.50)
Primary Codes ± 7 days	555	961	61.6 (57.6, 65.7)	99.1 (99.1, 99.2)	35.6 (33.2, 37.9)	99.7 (99.7, 99.7)	0.45 (0.41-0.48)
Primary Codes ± 30 days	555	907	63.2 (59.2, 67.3)	99.2 (99.2, 99.3)	38.7 (36.2, 41.2)	99.7 (99.7, 99.7)	0.48 (0.44-0.51)
Primary Codes ± 14 days - Excluding Patients with Prior Bleeding	153	311	66.0 (58.5, 73.5)	99.2 (99.1, 99.3)	32.5 (28.6, 36.3)	99.8 (99.8, 99.8)	0.43 (0.37-0.49)
Primary/Secondary Codes ± 14 days	555	1273	72.6 (68.9, 76.3)	98.8 (98.7, 98.9)	31.7 (29.9, 33.5)	99.8 (99.8, 99.8)	0.43 (0.41-0.46)
Primary/Secondary Codes ± 7 days	555	1273	72.1 (68.3, 75.8)	98.8 (98.7, 98.9)	31.4 (29.6, 33.2)	99.8 (99.8, 99.8)	0.43 (0.40-0.46)
Primary/Secondary Codes ± 30 days	555	1271	73.9 (70.2, 77.5)	98.8 (98.7, 98.9)	32.3 (30.5, 34.1)	99.8 (99.8, 99.8)	0.44 (0.41-0.47)
Primary/Secondary Codes ± 14 days - Excluding Patients with Prior Bleeding	153	400	77.1 (70.5, 83.8)	98.9 (98.8, 99.0)	29.5 (26.5, 32.5)	99.9 (99.8, 99.9)	0.42 (0.37-0.47)
**Gastrointestinal Bleeding Events**							
Primary Codes ± 14 days (primary analysis)	3739	5188	55.9	95.5	40.3	97.6	0.43 (0.42-0.45)

			(54.3, 57.5)	(95.4, 95.7)	(39.2, 41.3)	(97.5, 97.7)	
Primary Codes ± 7 days	3748	5193	54.9 (53.3, 56.5)	95.5 (95.3, 95.6)	39.7 (38.6, 40.7)	97.5 (97.4, 97.6)	0.43 (0.41-0.44)
Primary Codes ± 30 days	3725	5182	57.0 (55.4, 58.6)	95.6 (95.4, 95.7)	41.0 (39.9, 42.1)	97.6 (97.5, 97.7)	0.44 (0.43-0.46)
Primary Codes ± 14 days + RBC Transfusion**	3738	8841	66.5 (65.0, 68.1)	91.0 (90.8, 91.2)	28.1 (27.5, 28.8)	98.1 (98.0, 98.2)	0.35 (0.34-0.36)
Primary Codes ± 14 days - Excluding Patients with Prior Bleeding	419	630	51.8 (47.0, 56.6)	98.4 (98.2, 98.6)	34.4 (31.4, 37.5)	99.2 (99.1, 99.3)	0.40 (0.36-0.44)
Primary/Secondary Codes ± 14 days	3743	8553	72.7 (71.2, 74.1)	91.7 (91.5, 91.9)	31.8 (31.1, 32.5)	98.4 (98.4, 98.5)	0.40 (0.39-0.41)
Primary/Secondary Codes ± 7 days	3750	8561	71.6 (70.2, 73.0)	91.6 (91.4, 91.83)	31.4 (30.7, 32.1)	98.4 (98.3, 98.5)	0.39 (0.38-0.40)
Primary/Secondary Codes ± 30 days	3732	8543	73.8 (72.4, 75.2)	91.7 (91.5, 91.9)	32.2 (31.6, 32.9)	98.5 (98.4, 98.6)	0.41 (0.40-0.42)
Primary/Secondary Codes ± 14 days + RBC Transfusion**	3744	11869	76.0 (74.7, 77.4)	87.4 (87.2, 87.7)	24.0 (23.5, 24.5)	98.6 (98.5, 98.7)	0.31 (0.30-0.32)
Primary/Secondary Codes ± 14 days - Excluding Patients with Prior Bleeding	421	1230	74.4 (70.2, 78.5)	96.5 (96.2, 96.7)	25.5 (23.8, 27.1)	99.6 (99.5, 99.6)	0.36 (0.33-0.39)
**Other Major Bleeding Events**							
Primary Codes ± 14 days (primary analysis)	967	1974	20.4 (17.8, 22.9)	97.5 (97.4, 97.59)	10.0 (8.8, 11.2)	98.9 (98.9, 98.9)	0.12 (0.10-0.13)
Primary Codes ± 7 days	968	1974	19.6 (17.1, 22.1)	97.5 (97.4, 97.6)	9.6 (8.5, 10.8)	98.9 (98.9, 98.9)	0.11 (0.10-0.13)
Primary Codes ± 30 days	963	1973	20.8 (18.2, 23.3)	97.5 (97.4, 97.6)	10.1 (8.9, 11.3)	98.9 (98.9, 98.9)	0.12 (0.10-0.14)
Primary Codes ± 14 days + RBC Transfusion*	964	6972	33.8 (30.8, 36.8)	90.8 (90.6, 91.0)	4.7 (4.3, 5.1)	99.0 (99.0, 99.1)	0.06 (0.05-0.07)
Primary/Secondary Codes ± 14 days	960	13958	50.2 (47.1, 53.4)	81.8 (81.6, 82.1)	3.5 (3.2, 3.7)	99.2 (99.2, 99.3)	0.04 (0.04-0.05)
Primary/Secondary Codes ± 7 days	964	13959	47.9 (44.8, 51.1)	81.8 (81.5, 82.1)	3.3 (3.1, 3.5)	99.2 (99.1, 99.2)	0.04 (0.03-0.04)
Primary/Secondary Codes ± 30 days	953	14175	52.6 (49.4, 55.7)	81.6 (81.3, 81.9)	3.5 (3.3, 3.8)	99.3 (99.2, 99.3)	0.04 (0.04-0.05)
Primary/Secondary Codes ± 14 days + RBC Transfusion**	959	15639	53.7 (50.6, 56.9)	79.9 (79.6, 80.2)	3.3 (3.1, 3.5)	99.3 (99.2, 99.3)	0.04 (0.03-0.04)

*The total number of events in the LAAO Registry and in claims data can vary with similar definitions due to differences in how overlapping events are accounted for (see Methods).

**Analyses included any hospitalization with an RBC transfusion ICD-10-PCS code transfusion as an event

Supplemental Table 8. Stroke Events in Claims Data Compared to Registry Data Based on LAAO Hospital*

**Ischemic Stroke - Primary Diagnosis Codes with Claims Events only at LAAO Hospital**					
**Present in Registry (N)****	**Present in Claims Data* (N)**			**Specificity, % (95% CI)**	**PPV, % (95% CI)**
	**Yes**	**No**	**Total**		
**Yes**	460	538	998	99.5 (99.4-99.5)	55.8 (52.7-58.8)
**No**	365	69849	70214		
**Total**	825	70387	71212		
**Ischemic Stroke - Primary Diagnosis Codes with Claims Events only at non-LAAO Hospitals**					
**Yes**	154	844	998	99.7 (99.6-99.7)	39.7 (35.1-44.3)
**No**	234	69967	70201		
**Total**	388	70811	71199		
**Ischemic Stroke - Primary & Secondary Diagnosis Codes with Claims Events only at LAAO Hospital**					
**Yes**	535	463	998	99.2 (99.2-99.3)	50.2 (47.6-52.8)
**No**	531	69703	70244		
**Total**	1066	70166	71232		
**Ischemic Stroke - Primary & Secondary Diagnosis Codes with Claims Events only at non-LAAO Hospitals**					
**Yes**	182	815	997	99.5 (99.5-99.6)	34.6 (30.8-38.4)
**No**	344	69804	70208		
**Total**	526	70679	71205		
**Hemorrhagic Stroke - Primary Diagnosis Codes with Claims Events only at LAAO Hospital**					
**Present in Registry (N)[a]**	**Present in Claims Data (N)**			**Specificity, %**	**PPV, %**
	**Yes**	**No**	**Total**		
**Yes**	81	160	241	99.9 (99.9-99.9)	52.9 (45.7-60.2)
**No**	72	70803	70875		
**Total**	153	70963	71116		
**Hemorrhagic Stroke - Primary Diagnosis Codes with Claims Events only at non-LAAO Hospitals**					

**Yes**	24	217	241	>99.9 (>99.9 - >99.9)	46.2 (33.0-59.3)
**No**	28	70841	70869		
**Total**	52	71058	71110		
**Hemorrhagic Stroke - Primary & Secondary Diagnosis Codes with Claims Events only at LAAO Hospital**					
**Yes**	100	141	241	99.8 (99.7-99.8)	37.5 (32.5-42.5)
**No**	167	70714	70881		
**Total**	267	70855	71122		
**Hemorrhagic Stroke - Primary & Secondary Diagnosis Codes with Claims Events only at non-LAAO Hospitals**					
**Yes**	33	208	241	99.9 (99.9-99.9)	28.9 (21.0-36.9)
**No**	81	70799	70880		
**Total**	114	71007	71121		

*Events reported in the LAAO Registry do not specify at which hospital the event occurred. The location of events in claims data were determined based on a unique hospital identifier.

**The total number of events in the LAAO Registry and in claims data can vary with similar definitions due to differences in how overlapping events are accounted for (see Methods).

Supplemental Table 9. Stroke Events in Claims Data Compared to Registry Data at University and Private/Community Hospitals*

**Ischemic Stroke - Primary Diagnosis Codes with Claims Events only at University Hospital**					
**Present in Registry (N)****	**Present in Claims Data* (N)**			**Specificity, %**	**PPV, %**
	**Yes**	**No**	**Total**		
**Yes**	86	41	127	99.0 (99.9-99.2)	45.0 (39.5-50.6)
**No**	105	10767	10872		
**Total**	191	10808	10999		
**Ischemic Stroke - Primary Diagnosis Codes with Claims Events only at Private/Community Hospitals**					
**Yes**	511	343	854	99.2 (99.1-99.3)	51.7 (49.1-54.3)
**No**	478	57416	57894		
**Total**	989	57759	58748		
**Ischemic Stroke - Primary & Secondary Diagnosis Codes with Claims Events only at University Hospital**					
**Yes**	92	35	127	98.6 (98.3-98.8)	36.8 (32.4-41.2)
**No**	158	10723	10881		
**Total**	250	10758	11008		
**Ischemic Stroke - Primary & Secondary Diagnosis Codes with Claims Events only at Private/Community Hospitals**					
**Yes**	598	256	854	98.8 (98.7-98.9)	46.3 (44.2-48.5)
**No**	693	57229	57922		
**Total**	1291	57485	58776		
**Hemorrhagic Stroke - Primary Diagnosis Codes with Claims Events only at University Hospital**					
**Present in Registry (N)[a]**	**Present in Claims Data (N)**			**Specificity, %**	**PPV, %**
	**Yes**	**No**	**Total**		
**Yes**	22	22	44	99.8 (99.7-99.9)	52.4 (39.2-65.6)
**No**	20	10925	10945		
**Total**	42	10947	10989		
**Hemorrhagic Stroke - Primary Diagnosis Codes with Claims Events only at Private/Community Hospitals**					

**Yes**	77	113	190	99.9 (99.8-99.9)	50.0 (43.0-57.1)
**No**	77	58361	58438		
**Total**	154	58474	58628		
**Hemorrhagic Stroke - Primary & Secondary Diagnosis Codes with Claims Events only at University Hospital**					
**Yes**	29	15	44	99.6 (99.5-99.7)	38.2 (29.8-46.6)
**No**	47	10904	10951		
**Total**	76	10919	10995		
**Hemorrhagic Stroke - Primary & Secondary Diagnosis Codes with Claims Events only at Private/Community Hospitals**					
**Yes**	97	93	190	99.7 (99.6-99.7)	33.7 (29.3-38.1)
**No**	191	58260	58451		
**Total**	288	58353	58641		

*Events reported in the LAAO Registry do not specify at which hospital the event occurred. The location of events in claims data were determined based on a unique hospital identifier.

**The total number of events in the LAAO Registry and in claims data can vary with similar definitions due to differences in how overlapping events are accounted for (see Methods).

Supplemental Table 10. Bleeding Events in Claims Data Compared to Registry Data Based on LAAO Hospital*

Present in Registry (N)[a]	Present in Claims Data** (N)			Specificity, %	PPV, %
	Yes	No	Total		
**All Major Bleeding Events – Primary Diagnosis Codes with Claims Events only at LAAO Hospital**					
**Yes**	2214	3042	5256	95.4 (95.3-95.6)	41.6 (40.5-42.7)
**No**	3110	64665	67775		
**Total**	5324	67707	73031		
**All Major Bleeding Events – Primary Diagnosis Codes with Claims Events only at non-LAAO Hospitals**					
**Yes**	878	4383	5261	97.1 (97.0-97.3)	31.2 (29.6-32.8)
**No**	1935	65423	67358		
**Total**	2813	69806	72619		
**All Major Bleeding Events – Primary & Secondary Diagnosis Codes with Claims Events only at LAAO Hospital**					
**Yes**	2843	2398	5241	87.6 (87.4-87.9)	24.8 (24.2-25.4)
**No**	8627	61164	69791		
**Total**	11470	63562	75032		
**All Major Bleeding Events – Primary & Secondary Diagnosis Codes with Claims Events only at non-LAAO Hospitals**					
**Yes**	1124	4135	5259	92.3 (92.1-92.5)	17.5 (16.7-18.3)
**No**	5297	63188	68485		
**Total**	6421	67323	73744		

*Events reported in the LAAO Registry do not specify at which hospital the event occurred. The location of events in claims data were determined based on a unique hospital identifier.

**The total number of events in the LAAO Registry and in claims data can vary with similar definitions due to differences in how overlapping events are accounted for (see Methods).

Supplemental Table 11. Bleeding Events in Claims Data Compared to Registry Data at University and Private/Community Hospitals *

**All Major Bleeding Events – Primary Diagnosis Codes with Claims Events only at University Hospital**					
**Present in Registry (N)[a]**	**Present in Claims Data** (N)**			**Specificity, %**	**PPV, %**
	**Yes**	**No**	**Total**		
**Yes**	478	393	871	90.7 (90.2-91.3)	32.7 (30.8-34.5)
**No**	985	9648	10633		
**Total**	1463	10041	11504		
**All Major Bleeding Events – Primary Diagnosis Codes with Claims Events only at Private/Community Hospitals**					
**Yes**	2457	1806	4263	93.0 (92.8-93.2)	38.4 (37.5-39.3)
**No**	3944	52432	56376		
**Total**	6401	54238	60639		
**All Major Bleeding Events – Primary & Secondary Diagnosis Codes with Claims Events only at University Hospital**					
**Yes**	610	257	867	77.4 (76.7-78.2)	19.3 (18.4-20.2)
**No**	2552	8752	11304		
**Total**	3162	9009	12171		
**All Major Bleeding Events – Primary & Secondary Diagnosis Codes with Claims Events only at Private/Community Hospitals**					
**Yes**	3098	1158	4256	81.8 (81.5-82.1)	22.3 (21.9-22.8)
**No**	10768	48319	59087		
**Total**	13866	49477	63343		

*Events reported in the LAAO Registry do not specify at which hospital the event occurred. The location of events in claims data were determined based on a unique hospital identifier.

**The total number of events in the LAAO Registry and in claims data can vary with similar definitions due to differences in how overlapping events are accounted for (see Methods).

Supplemental Table 12. Two-Year Incidence of Clinical Events Following LAAO Hospitalization Discharge in Claims vs. Registry Data

Clinical Event	Two-Year Cumulative Incidence in Claims Data, %	Two-Year Cumulative Incidence in Registry Data, %	HR [95% CI]	P Value
All Stroke	2.84 (2.69-2.99)	2.79 (2.63-2.95)	1.02 [0.94-1.10]	0.635
Ischemic Stroke	2.47 (2.32-2.64)	2.15 (2.02-2.30)	1.15 [1.05-1.26]	0.002
Hemorrhagic Stroke	0.39 (0.33-0.44)	0.48 (0.41-0.56)	0.81 [0.66-0.98]	0.030
All Major Bleeding	10.65 (10.39-10.91)	7.52 (7.30-7.74)	1.44 [1.39-1.50]	<0.001
Intracranial Hemorrhage	1.56 (1.47-1.66)	1.08 (0.99-1.17)	1.46 [1.30-1.63]	<0.001
Gastrointestinal Bleeding	6.77 (6.56-6.99)	5.21 (5.04-5.39)	1.31 [1.25-1.37]	<0.001
Other Major Bleeding	3.06 (2.92-3.21)	1.49 (1.39-1.60)	2.07 [1.90-2.25]	<0.001

Incidence rates were determined using Kaplan-Meier methods, and hazard ratios using the data source indicator (registry or claims) were determined using unadjusted competing risk models with Cox regression. P values represent differences between registry and claims using an indicator for data source in the models.

Supplemental Table 13. Other Major Bleeding Events in Claims Data Compared to Registry Data in Early vs. Late Follow-Up

Time of Other Major Bleeding Event	No. of Events in Registry	No. of Events in Claims	Sensitivity,% (95% CI)	PPV,% (95% CI)	Kappa Coefficient (95% CI)
Early Follow-Up (1-365 Days Post-Discharge)	853	1731	20.8 (18.0-23.5)	10.2 (8.9-11.5)	0.11 (0.09-0.13)
Late Follow-Up (366-720 Days Post-Discharge)	114	243	17.5 (10.6-24.5)	8.2 (5.1-11.4)	0.11 (0.06-0.15)

In analyses evaluating cumulative incidence for registry vs. claims, other major bleeding events did not meet the assumption of proportional hazards ($p < 0.001$). All other individual events met the assumption for proportional hazards ($p > 0.05$).

Supplemental Table 14. All-Cause Mortality in LAAO Registry Compared to CMS Data

Present in Registry (n)	Present in Claims Data (n)			Sensitivity, % (95% CI)	Specificity, % (95% CI)	PPV, % (95% CI)	NPV, % (95% CI)	Kappa Coefficient (95% CI)
	**Yes**	**No**	**Total**					
**Yes**	895	1	896	93.0 (91.4-94.6)	>99.9 (>99.9 - >99.9)	99.9 (99.7 - >99.9)	99.5 (99.4-99.6)	0.96 (0.95-0.97)
**No**	67	14451	14518					
**Total**	962	14452	15414					

Analyses were based on all-cause mortality using death in CMS data as the reference standard.

Supplemental Figure 1. Cohort for Comparison of Claims Data to LAAO Registry-Reported Events

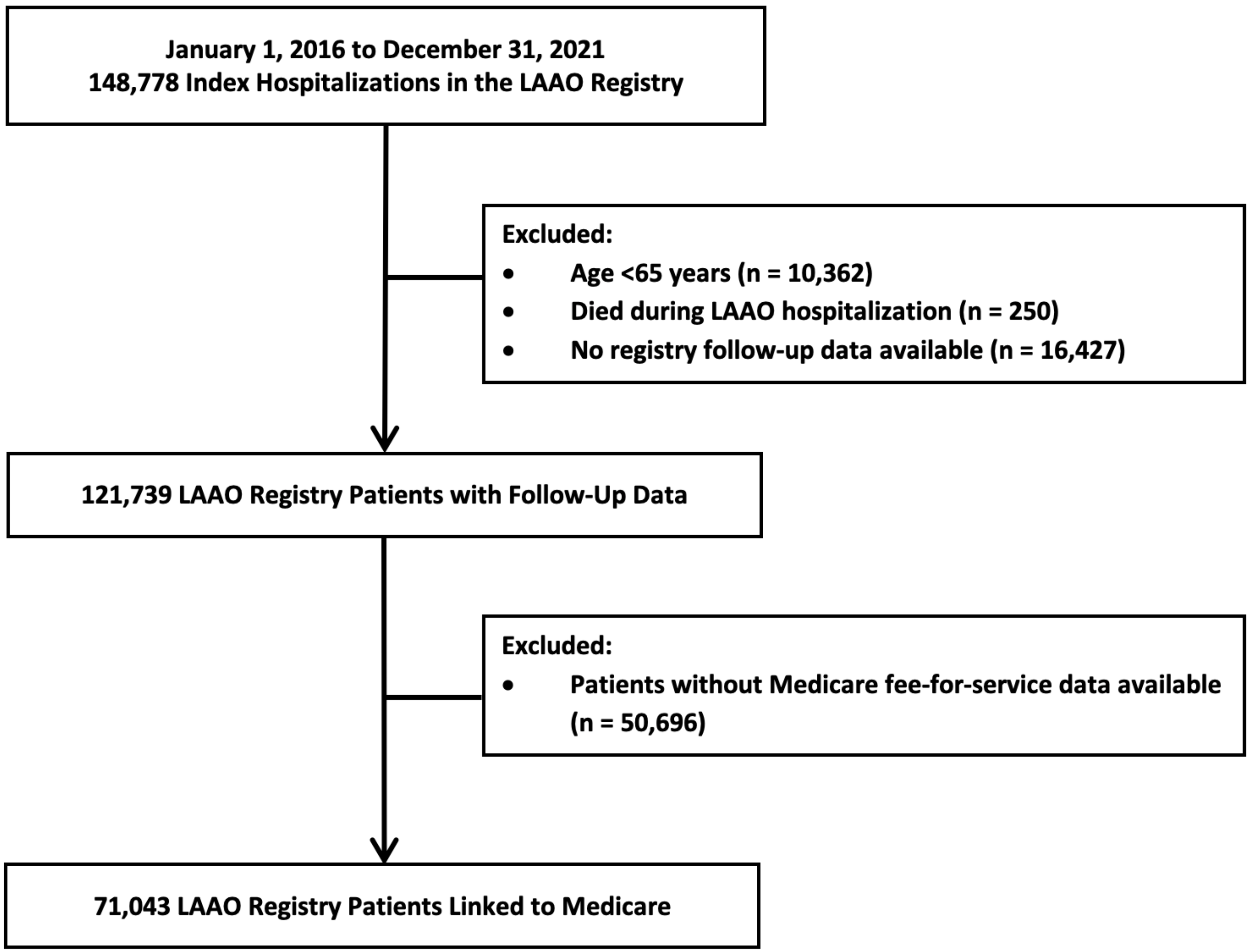

STARD Checklist

Section & Topic	No	Item	Reported on page #
**TITLE OR ABSTRACT**			
	**1**	Identification as a study of diagnostic accuracy using at least one measure of accuracy (such as sensitivity, specificity, predictive values, or AUC)	2
**ABSTRACT**			
	**2**	Structured summary of study design, methods, results, and conclusions (for specific guidance, see STARD for Abstracts)	2
**INTRODUCTION**			6-7
	**3**	Scientific and clinical background, including the intended use and clinical role of the index test	
	**4**	Study objectives and hypotheses	
**METHODS**			
Study design	**5**	Whether data collection was planned before the index test and reference standard were performed (prospective study) or after (retrospective study)	7-8
Participants	**6**	Eligibility criteria	8
	**7**	On what basis potentially eligible participants were identified (such as symptoms, results from previous tests, inclusion in registry)	8
	**8**	Where and when potentially eligible participants were identified (setting, location and dates)	8
	**9**	Whether participants formed a consecutive, random or convenience series	8
Test methods	**10a**	Index test, in sufficient detail to allow replication	9-12
	**10b**	Reference standard, in sufficient detail to allow replication	7-9
	**11**	Rationale for choosing the reference standard (if alternatives exist)	9
	**12a**	Definition of and rationale for test positivity cut-offs or result categories of the index test, distinguishing pre-specified from exploratory	9-12
	**12b**	Definition of and rationale for test positivity cut-offs or result categories of the reference standard, distinguishing pre-specified from exploratory	9-12
	**13a**	Whether clinical information and reference standard results were available to the performers/readers of the index test	9-12
	**13b**	Whether clinical information and index test results were available to the assessors of the reference standard	9-12
Analysis	**14**	Methods for estimating or comparing measures of diagnostic accuracy	10-12
	**15**	How indeterminate index test or reference standard results were handled	10-11
	**16**	How missing data on the index test and reference standard were handled	10-11
	**17**	Any analyses of variability in diagnostic accuracy, distinguishing pre-specified from exploratory	10-11
	**18**	Intended sample size and how it was determined	8
**RESULTS**			
Participants	**19**	Flow of participants, using a diagram	8 + Supplement
	**20**	Baseline demographic and clinical characteristics of participants	33, Table 1
	**21a**	Distribution of severity of disease in those with the target condition	33, Table 1
	**21b**	Distribution of alternative diagnoses in those without the target condition	33, Table 1
	**22**	Time interval and any clinical interventions between index test and reference standard	7-8
Test results	**23**	Cross tabulation of the index test results (or their distribution) by the results of the reference standard	Tables 2-3, Figures 1-2, Supplement
	**24**	Estimates of diagnostic accuracy and their precision (such as 95% confidence intervals)	Tables 2-3, Supplement
	**25**	Any adverse events from performing the index test or the reference standard	Tables 2-3, Supplement
**DISCUSSION**			
	**26**	Study limitations, including sources of potential bias, statistical uncertainty, and generalisability	20-21
	**27**	Implications for practice, including the intended use and clinical role of the index test	19-20
**OTHER INFORMATION**			

	**28**	Registration number and name of registry	7
	**29**	Where the full study protocol can be accessed	7-12 + Supplement
	**30**	Sources of funding and other support; role of funders	5
